# Severe COVID-19 patients display hyper-activated NK cells and NK cell-platelet aggregates

**DOI:** 10.3389/fimmu.2022.861251

**Published:** 2022-10-05

**Authors:** Bert Malengier-Devlies, Jessica Filtjens, Kourosh Ahmadzadeh, Bram Boeckx, Jessica Vandenhaute, Amber De Visscher, Eline Bernaerts, Tania Mitera, Cato Jacobs, Lore Vanderbeke, Pierre Van Mol, Yannick Van Herck, Greet Hermans, Philippe Meersseman, Alexander Wilmer, Mieke Gouwy, Abhishek D. Garg, Stephanie Humblet-Baron, Frederik De Smet, Kimberly Martinod, Els Wauters, Paul Proost, Carine Wouters, Georges Leclercq, Diether Lambrechts, Joost Wauters, Patrick Matthys

**Affiliations:** ^1^ Laboratory of Immunobiology, Department of Microbiology, Immunology and Transplantation, Rega Institute, KU Leuven, Leuven, Belgium; ^2^ Laboratory of Translational Genetics, Department of Human Genetics, VIB-KU Leuven, Leuven, Belgium; ^3^ Laboratory for Clinical Infectious and Inflammatory Disorders, Department of Microbiology, Immunology and Transplantation, KU Leuven, Leuven, Belgium; ^4^ Laboratory of Clinical Bacteriology and Mycology, Department of Microbiology, Immunology and Transplantation, KU Leuven, Leuven, Belgium; ^5^ Laboratory of Experimental Oncology, Department of Oncology, KU Leuven, Leuven, Belgium; ^6^ Laboratory of Intensive Care Medicine, Department of Cellular and Molecular Medicine, KU Leuven, Leuven, Belgium; ^7^ Laboratory of Molecular Immunology, Department of Microbiology, Immunology and Transplantation, Rega Institute, KU Leuven, Leuven, Belgium; ^8^ Laboratory for Cell Stress & Immunity (CSI), Department of Cellular and Molecular Medicine (CMM), KU Leuven, Leuven, Belgium; ^9^ Adaptive Immunology, Department of Microbiology, Immunology and Transplantation, KU Leuven, Leuven, Belgium; ^10^ Laboratory for Precision Cancer Medicine, Translational Cell and Tissue Research, Department of Imaging & Pathology, KU Leuven, Leuven, Belgium; ^11^ Centre for Molecular and Vascular Biology, Department of Cardiovascular Sciences, KU Leuven, Leuven, Belgium; ^12^ Laboratory of Respiratory Diseases and Thoracic Surgery (BREATHE), Department of Chronic Diseases and Metabolism, KU Leuven, Leuven, Belgium; ^13^ Laboratory of Experimental Immunology, Department of Diagnostic Sciences, Ghent University, Ghent, Belgium

**Keywords:** COVID-19, NK cells, cytokines, cytotoxicity, platelet aggregates

## Abstract

COVID-19 is characterised by a broad spectrum of clinical and pathological features. Natural killer (NK) cells play an important role in innate immune responses to viral infections. Here, we analysed the phenotype and activity of NK cells in the blood of COVID-19 patients using flow cytometry, single-cell RNA-sequencing (scRNA-seq), and a cytotoxic killing assay. In the plasma of patients, we quantified the main cytokines and chemokines. Our cohort comprises COVID-19 patients hospitalised in a low-care ward unit (WARD), patients with severe COVID-19 disease symptoms hospitalised in intensive care units (ICU), and post-COVID-19 patients, who were discharged from hospital six weeks earlier. NK cells from hospitalised COVID-19 patients displayed an activated phenotype with substantial differences between WARD and ICU patients and the timing when samples were taken post-onset of symptoms. While NK cells from COVID-19 patients at an early stage of infection showed increased expression of the cytotoxic molecules perforin and granzyme A and B, NK cells from patients at later stages of COVID-19 presented enhanced levels of IFN-γ and TNF-α which were measured *ex vivo* in the absence of usual *in vitro* stimulation. These activated NK cells were phenotyped as CD49a^+^CD69a^+^CD107a^+^ cells, and their emergence in patients correlated to the number of neutrophils, and plasma IL-15, a key cytokine in NK cell activation. Despite lower amounts of cytotoxic molecules in NK cells of patients with severe symptoms, majority of COVID-19 patients displayed a normal cytotoxic killing of Raji tumour target cells. *In vitro* stimulation of patients blood cells by IL-12+IL-18 revealed a defective IFN-γ production in NK cells of ICU patients only, indicative of an exhausted phenotype. ScRNA-seq revealed, predominantly in patients with severe COVID-19 disease symptoms, the emergence of an NK cell subset with a platelet gene signature that we identified by flow and imaging cytometry as aggregates of NK cells with CD42a^+^CD62P^+^ activated platelets. Post-COVID-19 patients show slow recovery of NK cell frequencies and phenotype. Our study points to substantial changes in NK cell phenotype during COVID-19 disease and forms a basis to explore the contribution of platelet-NK cell aggregates to antiviral immunity against SARS-CoV-2 and disease pathology.

## Introduction

SARS-CoV-2 is a single-stranded RNA virus causing COVID-19 disease. COVID-19 is characterised by a broad spectrum of disease symptoms and severity. Patients with mild symptoms generally present with fever, non-productive cough, dyspnea, and fatigue. Around 20% develop severe disease symptoms, requiring hospitalisation when pneumonia, acute respiratory distress syndrome (ARDS), and multiorgan failure develop (1–3). About 5% of COVID-19 patients necessitate intensive care (4).

A large number of studies have contributed to a better understanding of the pathogenesis underlying COVID-19. These studies focused on the molecular aspects of virus replication and the pathologies associated with COVID-19. Concerning immune pathology, there is abundant evidence and consensus that immune dysfunction is a hallmark of severe COVID-19, and these include hypercytokinemia, neutrophil hyperactivation, monocyte dysfunction, immunothrombosis, and a defective type I interferon response (5–8). In the present study, we performed a comprehensive analysis of natural killer (NK) cells in COVID-19. NK cells are innate lymphocytes with potent cytotoxic machinery (i.e. the release of perforin and granzymes), which enables them to kill infected and neoplastic target cells within hours (9, 10). The activation of NK cells depends on signals received by their activating and inhibitory receptors which are triggered by ligands on NK cell target cells, such as infected cells and tumour cells. Because chronically activated lymphocytes and monocytes express NK-cell ligands, they can also be targeted by NK cells. This less recognised characteristic of NK cells has become especially evident in hemophagocytic lymphohistiocytosis (HLH) and macrophage activation syndrome (MAS), both hyperinflammatory cytokine storm syndromes resulting from cytotoxic defects in NK cells and T cells (11–13). Another way of NK cell activation is through their Fc receptor CD16, enabling them to detect and kill antibody-coated target cells *via* antibody-dependent cell-mediated cytotoxicity (ADCC). In addition to their cytolytic activities, NK cells vigorously produce cytokines, such as IFN-γ and TNF-α, and chemokines, such as CCL2, CCL3, CCL4, CCL5, and CXCL8, thereby further stimulating innate and adaptive immune pathays (14). Thus, during infection, NK cell activation must be carefully regulated to eradicate infected cells and to avoid a hyper-inflammatory syndrome.

Human peripheral blood NK cells have long been classified into two functionally distinct subsets. CD56^dim^ cells represent the largest circulating NK cell subset expressing high levels of CD16 and displaying profound cytolytic activities. CD56^bright^ cells, on the other hand, are more abundant in secondary lymphoid organs, are less cytotoxic, and secrete high levels of cytokines and chemokines. Expanding from this traditional view of two subsets, it is now clear that the spectrum of human NK cell diversity is broader (15). During SARS-CoV-2 infection, a reduced number of total peripheral blood NK cells was reported in patients with mild and severe disease (16–22). In contrast, at the site of infection, in the bronchoalveolar lavage fluid (BALF), increased numbers of NK cells were found (23–25). Some studies also reported that NK cells can expand to adaptive NK cells, which represent a terminally differentiated subset of NK cells defined by increased expression of NKG2C and CD57 (26, 27). Regarding the cytotoxic activity of NK cells in COVID-19, functional impairment, which is founded by either indirect observations such as decline of perforin levels, increase in exhaustion markers, or decreased degranulation, as measured by CD107a expression when PBMCs from COVID-19 patients are cocultured with tumour target cells (19, 28–31). Some studies have used an *in vitro* cytotoxic killing assay for the assessment of cytotoxicity. In this regard, Demaria et al. found that peripheral blood mononuclear cell (PBMCs) from COVID-19 patients retained their cytotoxic functions, as evident from the lysis of tumour target cells (22). Other studies reported that NK cells of (severe) COVID-19 patients display impaired lysis of K562 tumour cells and impaired antiviral activity towards SARS-CoV-2-infected Vero E6 cells (31–33).

In the present study, we comprehensively analysed the NK cell phenotype in COVID-19 patients while focusing on the cytolytic and cytokine-producing activities of NK cells. We took advantage of our established transdisciplinary COVID-19 Advanced Genetic and Immunologic Sampling (CONTAGIOUS) consortium for which we have collected and analysed a large number of PBMCs and plasma samples of COVID-19 patients, hospitalised both in a general low-care ward unit (WARD) as in intensive care units (ICU) of the University Hospital Leuven, Belgium. In our cohort, we also included samples from post-COVID-19 patients, collected six weeks after hospital discharge.

We report differences in NK cell subsets and phenotypes from COVID-19 patients in WARD and ICU. We found that during COVID-19 progression, *ex vivo* NK cells, thus without deliberate *in vitro* stimulation, display a skewed cytokine-producing profile. While the majority of COVID-19 patients exhibit normal cytotoxic activity against Raji tumour target cells, some ICU patients failed to demonstrate cytolysis, also in an ADCC assay. In addition, NK cells obtained from severe COVID-19 patients with a long disease duration were phenotyped with a low expression of cytotoxic molecules and had a lower *in vitro* cytokine production upon IL-12+IL-18 stimulation, all indicative of an exhausted phenotype. Analysis of blood samples from patients six weeks after hospital discharge shows a slow recovery of the NK cytotoxic molecules. Furthermore, single-cell RNA-sequencing (scRNA-seq) on PBMCs revealed, predominantly in ICU patients, the emergence of an NK cell subset with a platelet gene signature that we identified by flow and imaging cytometry as aggregates of NK cells and activated platelets.

## Methods

### Patient material

Samples of patients infected with SARS-CoV-2 and healthy controls (HCs) were collected between March 19^th^ and June 7^th^, 2020 in the University Hospitals Leuven as part of the CONTAGIOUS consortium. SARS-CoV-2 samples were classified in 36 WARD patients and 61 ICU patients, of which we collected samples at different time points during disease. Follow-up samples were collected from 8 WARD patients and 20 ICU patients, six weeks after discharge from the hospital (post-COVID-19 patients). COVID-19 was defined as a positive qRT-PCR (n=87) on a respiratory sample and/or CT imaging highly suggestive of COVID-19 (n=8, no qRT-PCR was performed) compatible with COVID-19. Control samples were derived from hospital staff with negative COVID-19 serology (n=18). The demographic and disease characteristics are listed in **Supplementary Table 1**. Patients comorbidities, non-COVID-related treatments and ethnicity are shown in **Supplementary Tables 2–4**. Demographic, clinical, treatment, and outcome data were obtained from the patient electronic medical records stored in the Research Electronic Data Capture Software REDCAP, Vanderbilt University.

Blood samples were collected in EDTA tubes, stored at 15°C, and processed within 3-6 hours. Separation of plasma and PBMCs was performed using a lymphocyte separation medium (LSM, MP Biomedicals), after which PBMCs were frozen in 10% dimethyl sulfoxide (Sigma) and stored in liquid nitrogen, while plasma was kept at -80°C until processing. Due to ethical constraints, a limited amount of blood was collected. Considering some patients had low blood cell counts, we have not always been able to perform a full flow cytometric analysis or cytotoxicity assay on all patient samples. In each analysis, the number of samples is indicated in the figure legends.

### Flow and imaging cytometry

Frozen PBMCs were thawed, washed with medium, and used for staining. Cells were incubated with FcR-block (Miltenyi Biotec) and stained with live/dead marker (Fixable Viability Stain 620, BD Biosciences). Next, cells were extracellularly stained with fluorochrome-conjugated antibodies against surface markers (**Supplementary Table 5**) and fixed with 4% paraformaldehyde. Staining for intracellular protein was performed with the Cytofix/Cytoperm kit (BD biosciences) according to the manufacturer’s instructions. Flow cytometry was performed on the BD LSR Fortessa X20, and data were analysed with FlowJo (LLC, V10). A representative gating strategy is shown in **Supplementary Figure 1**. NK-platelet aggregate complexes were studied using an ImageStream Imaging Flow Cytometer (Amnis Corporation). Flow cytometric data are presented in the three groups of patients (WARD, ICU and post-COVID-19) along with HCs. In addition, WARD and ICU patients were grouped according to the days post-onset of COVID-19 symptoms, i.e. day 0-10 (group 1), day 11-20 (group 2), day 21-30 (group 3), and >30 days (group 4).

### Detection of IFN-γ and TNF-α, *ex vivo* and upon *in vitro* stimulation with IL-12+IL-18

Levels of IFN-γ and TNF-α in NK cells were determined either *ex vivo* or upon *in vitro* stimulation with IL-12+IL-18. PBMCs used for *ex vivo* staining of cytokines were plated for 2 hours (without any *in vitro* stimulation) in complete RPMI containing Monensin (BD GolgiStop, 1:1500 dilution) and Brefeldin (BD GolgiPlug, 1:1000) at 37°C with 5% CO_2_. To measure the production of IFN-γ and TNF-α upon *in vitro* stimulation, PBMCs were cultured in medium alone or in presence of IL-12 (2 ng/ml, PeproTech) plus IL-18 (100 ng/ml, MBL) for 18 hours (with GolgiStop and GolgiPlug added during the last 4 hours of stimulation). Intracellular expression of IFN-γ and TNF-α, along with extracellular NK cell markers, was analysed by flow cytometry as described above.

### Single-cell RNA-sequencing

scRNA-seq was performed on PBMCs from 26 COVID-19 patients. 14 WARD patients and 12 ICU patients (all upon admission except for one at discharge) were included. Single-cell suspensions were converted to barcoded scRNA-seq libraries by using the Chromium Single Cell 5’ library and Gel Bead & Multiplex Kit from 10x Genomics. Captured cells were lysed and the released RNA was barcoded. Libraries were sequenced on an Illumina NovaSeq 6000 sequencer according to a paired-end reading strategy. Raw gene expression matrices were generated per sample by the Cell Ranger pipeline using the human reference version GRCh38. Additionally, we processed scRNA-seq data from Wilk et al. (17), including 6 HCs, 4 severe, and 4 moderate COVID patients. All datasets were analysed by the Seurat package and merged using Harmony. The R package Slingshot was used to explore pseudotime trajectories (34).

### Cytokine array

Chemokine and cytokine levels in plasma were assessed by Meso Scale Discovery (MSD) using the V-plex human cytokine 30-plex kit (Eotaxin, Eotaxin-3, GM-CSF, IFN-γ, IL-1α, IL-1β, IL-2, IL-4, IL-5, IL-6, IL-7, IL-8, IL-8 (HA), IL-10, IL-12/IL-23p40, IL-12p70, IL-13, IL-15, IL-16, IL-17A, IP-10 (CXCL10), MCP-1 (CCL2), MCP-4 (CCL13), MDC (CCL22), MIP-1α (CCL3), MIP-1β (CCL4), TARC (CCL17), TNF-α, TNF-β, VEGF-A), complemented with Human IL-1RA (V-plex), Human IL-18 (U-plex), and Human CXCL9 (R-plex) kits.

### NK cell cytotoxicity assay

Purified NK cells from PBMCs of COVID-19 patients were used in a chromium release assay to assess cytotoxicity. Briefly, NK cells from HCs and COVID-19 patients were enriched from PBMCs by magnetic negative selection using the human NK cell isolation kit (Stemcell Technologies), according to the manufacturer’s protocol. The purity of enriched cells was between 85-95% (data not shown). NK cells were stimulated overnight with IL-15 (20 ng/ml, PeproTech) before using them in the assay. NK cells were plated with Raji cells loaded with chromium 51 (^51^Cr) (Perkin Elmer) at effector:target (E:T) ratio’s of 3:1, 1:1, 0.3:1, and 0.1:1, respectively, in V-bottomed 96-well plates for 4h at 37°C. To determine ADCC, cells were incubated with rituximab (RTX), a monoclonal antibody that binds to CD20. All conditions were plated in duplicate. After incubation, the cell culture supernatant was transferred to a LumaPlate (Perkin Elmer) coated with a solid scintillator. The release of ^51^Cr was measured using the microplate scintillation counter (Perkin Elmer, Microbeta, 2450 Microplate counter). The percentage of specific lysis was calculated as [(experimental release – spontaneous release)/(maximal release – spontaneous release)] x 100. The maximal release was determined by adding a mixture of medium and soap to the target cells, while the spontaneous release was measured by adding medium alone without effector cells.

### Statistical analysis

Statistical analysis and plots were performed using R. Boxplots represent the median and 25^th^ to 75^th^ percentiles, and the whiskers denote the lowest and highest values. For the comparison of groups, P-values were obtained using Wilcoxon signed-rank tests, Bonferroni corrected for multiple comparisons. * Represent differences between indicated groups, # represent differences with HCs. */#p<0.05; **/##p<0.01; ***/###p<0.001; ****/####p<0.0001. For correlation statistics, Pearson correlation was applied.

### Study approval

All procedures were approved by the UZ Leuven Ethical Committee (protocol study number S63881). Informed consent was obtained from all individuals or their legal guardians according to the Declaration of Helsinki.

## Results

### Experimental design and clinical data of the COVID-19 cohort

**Figure 1:**
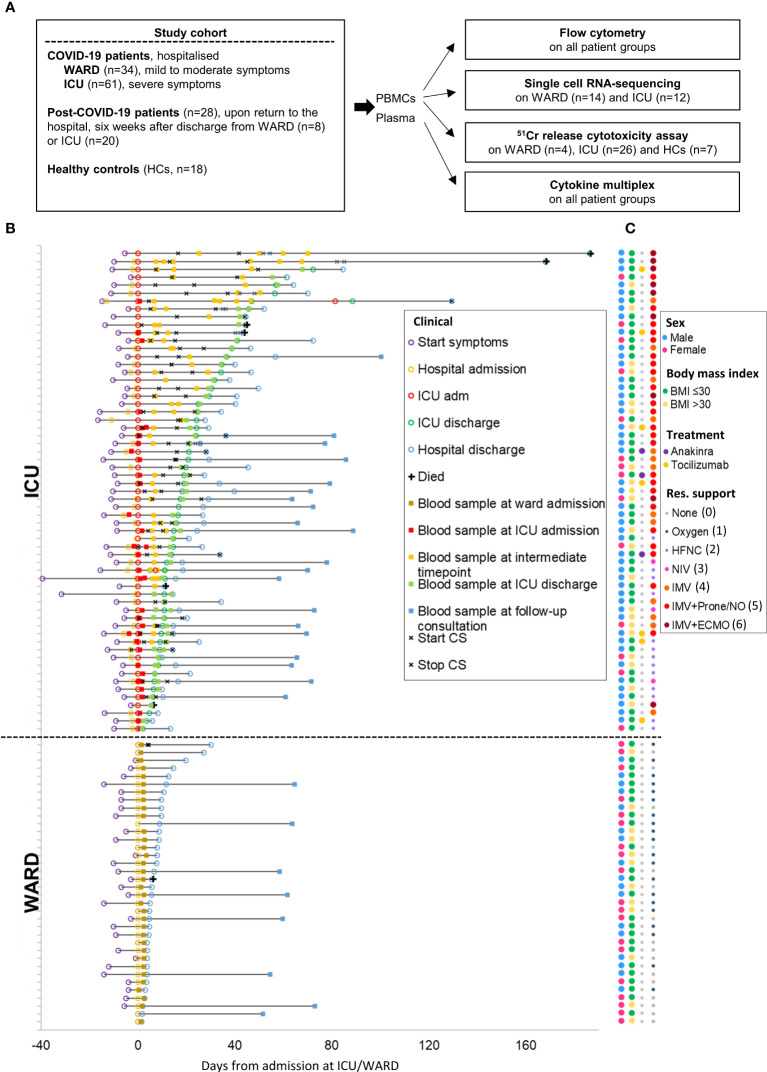
Study design and COVID-19 patient cohort. **(A)** Schematic overview of study design and the number of included patients. **(B)** Detailed timeline highlighting all clinical milestones according to their relative time (days) from each patient’s admission to ICU or WARD. Additionally, start and stop of corticosteroid (CS) treatment was shown. **(C)** Dot plot indicating the gender, body mass index (BMI), additional treatment, and maximal respiratory support. The respiratory support levels were classified from 0 to 6 [level 0 indicates no support, level 6 invasive mechanical ventilation (IMV) with extracorporeal membrane oxygenation (ECMO)]. Oxygen (level 1); HFNC, high flow nasal cannula (level 2); NIV, non-invasive ventilation (level 3); IMV (level 4) IMV + prone/NO, nitric oxide (level 5).

95 patients with COVID-19 were prospectively recruited after admission to the University Hospital Leuven, as part of the CONTAGIOUS trial (NCT04327570). Demographics and laboratory results at the time of blood sampling of COVID-19 patients are summarised in **Supplementary Tables 1–4**. As schematically presented in **Figure 1A**, recruited patients consisted of COVID-19 patients hospitalised at the WARD (n = 36) or ICU (n = 61). WARD patients had a mild to moderate clinical condition either receiving no respiratory support or oxygen *via* nasal cannula. ICU patients had a critical clinical condition receiving high flow oxygen support or mechanical ventilation. Blood samples were also collected from COVID-19 patients six weeks after discharge from the WARD (n = 8) or ICU (n = 20). These are referred to as post-COVID-19 patients. To study NK cells, PBMCs were isolated and analysed either by flow cytometry or scRNA-seq or were used to purify for NK cells to study their cytolytic activity (**Figure 1A**). In the flow cytometric analysis and NK cell activity assays, PBMCs from 18 HCs, with no prior diagnosis or symptoms of COVID-19 (see M&M), were included. In plasma from patients and HCs, cytokines were analysed by MSD multiplex. For each patient, a detailed timeline was generated displaying the onset of symptoms, time of hospital admission and discharge, time points of blood sampling, and glucocorticoids (CS) treatment (**Figure 1B**). **Figure 1C** additionally summarises gender, BMI, and provides details on respiratory levels, and additional treatment.

### Relative expansion of CD56^dim^ NK cells in COVID-19 patients during early phases of COVID-19

**Figure 2:**
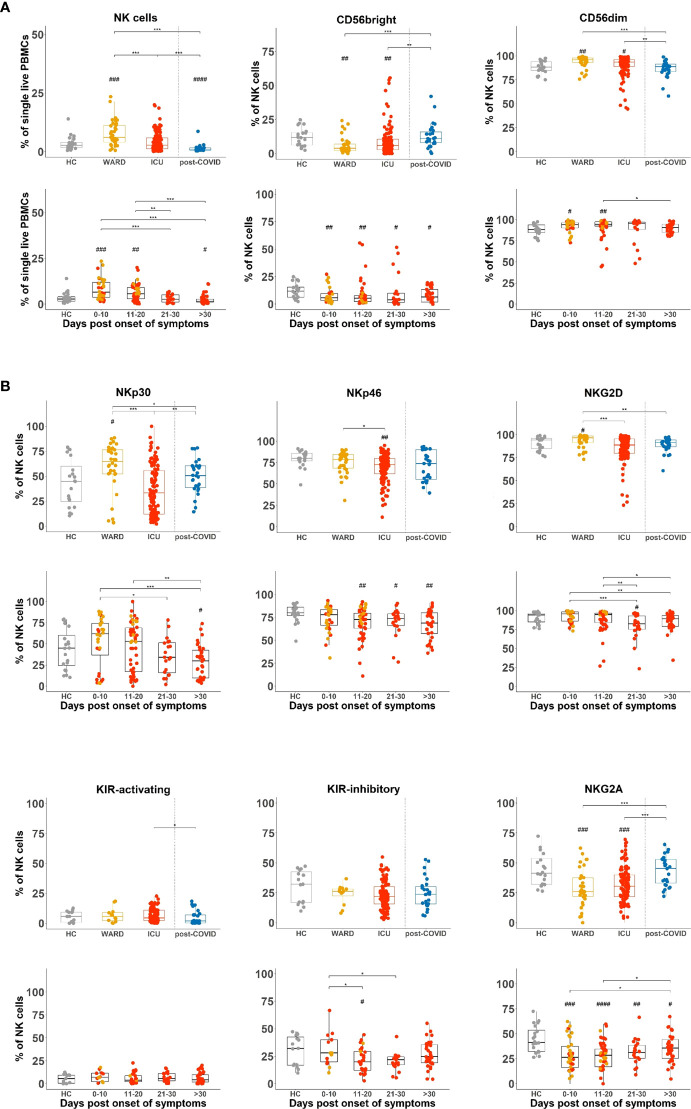
Flow cytometry reveals increased frequencies of CD56^dim^ NK cells in COVID-19 patients at early disease stages and with a different balance of activating and inhibitory NK cell receptors. **(A)** Percentages of total natural killer (NK) cells and NK cell subsets in peripheral blood mononuclear cells (PBMCs) of healthy controls (HCs) (n = 18), WARD (n = 34), ICU (n = 61), and post-COVID-19 patients (n = 28), upon return to the hospital six weeks after discharge. WARD (yellow) and ICU (red) samples were further subdivided based on the days post onset of COVID-19 symptoms. **(B)** Percentage expression of activating and inhibitory receptors on total NK cells of indicated experimental groups. Boxplots represent the median and upper and lower quartile, and the whiskers denote the lowest and highest values. Each symbol represents a single patient. For the comparison of groups, *p*-values were obtained using Wilcoxon signed-rank test with Bonferroni correction. * Represent differences between indicated groups, ^#^ represent differences with HCs. */^#^p<0.05; **/^##^p<0.01; ***/^###^p<0.001; ^####^p<0.0001.

NK cells were investigated by flow cytometry in our cohort of active and post-COVID-19 patients and further compared to HCs. Considering the differences in timing relative to the onset of COVID-19 symptoms in WARD and ICU patients, data are also presented after grouping blood samples from hospitalised patients (WARD + ICU) according to the days post onset of COVID-19 symptoms, i.e. day 0-10, day 11-20, day 21-30 and >30 days. SARS-CoV-2 infection is known to be associated with a dramatic drop in almost all blood leukocytes (except for neutrophils and plasmablast cells), as we and others previously reported (17, 35, 36). However, when the relative percentages of NK cells within total PBMCs were plotted, we found significantly increased frequencies of NK cells in WARD patients compared to HCs and ICU patients (**Figure 2A** upper panels). When NK cells were divided into CD56^bright^ and CD56^dim^ subsets, the CD56^dim^ subset was increased in WARD as well as in ICU patients, while consequently CD56^bright^ NK cells were decreased. Grouping of data according to days post onset of COVID-19 symptoms showed an increase in frequency of NK cells during the early phase of COVID-19 disease more specifically in samples taken from patients of 0-10 and 11-20 days post onset symptoms and revealed a significant decrease in NK cell numbers in blood of patients taken at >30 days post symptoms as compared to HCs (**Figure 2A** lower panels). NK cell numbers remained significantly lower in post-COVID-19 patients (**Figure 2A** upper panels).

### Differences in the balance of activating and inhibitory NK cell receptors in WARD and ICU patients

NK cell-mediated activities are tightly regulated by a balance of activating and inhibitory receptors to kill infected and malignant cells while leaving healthy cells intact. When we analysed the expression of activating (NKp30, NKp46, NKG2D, and activating KIRs) and inhibitory receptors (inhibitory KIRs and NKG2A) on total NK cells, we observed an increased frequency of NKp30+ and NKG2D+ NK cells in WARD patients (**Figure 2B** upper panels, MFI for NKp30 was significantly increased as shown in **Supplementary Figure 2**). In ICU patients, expansion of NKp30+ and NKG2D+ NK cells was not seen and levels were significantly lower than in WARD patients. NKp46 was decreased in ICU patients and partially restored in post-COVID patients. No differences could be detected in activating KIR and inhibitory KIR receptor expression. Analysis of the inhibitory NKG2A receptor showed a decreased percentage of NKG2A+ NK cells both in WARD and ICU patients, which was restored in the post-COVID group (**Figure 2B** upper panels, MFI of NKG2A was significantly lower in WARD patients only, **Supplementary Figure 2**). When days post onset of symptoms were taken into account, substantial differences between the four groups of patients were seen for NKp30 and NKG2D-expressing NK cells and were in general increased during early time point post onset of COVID-19 disease and decreased at later time points (**Figure 2B** lower panels). The decrease in frequency of NKG2A expressing NK cells was most evident during the early phase of COVID-19 disease. The same pattern of expression (increased frequency of NKp30+ or NKG2D+ NK cells in WARD and decreased percentage of NKG2A+ NK cells in WARD and ICU) was seen in both CD56^bright^ (NKG2D only) and CD56^dim^ NK cell subsets (**Supplementary Figure 3**).

In summary, the balance in activating and inhibitory NK cell receptors is shifted toward activation in WARD patients and in samples taken during the first 10 days post COVID-19 symptoms, but not in the other hospitalised patient groups. In post-COVID-19 patients, this balance is restored.

### During COVID-19 disease, NK cells undergo substantial changes in expression of cytotoxic molecules and levels of IFN-γ and TNF-α

**Figure 3:**
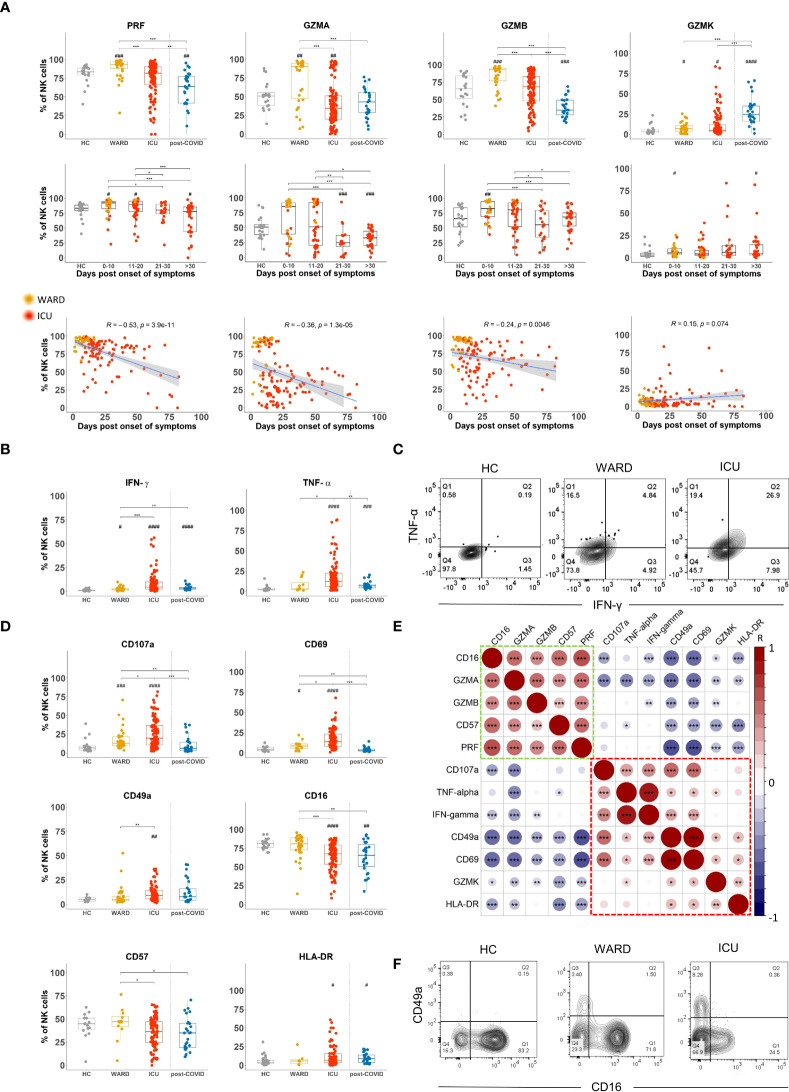
Shift in NK cell phenotype from a cytotoxic to a cytokine-producing profile in WARD and ICU and during the course of COVID-19 disease **(A)** Percentage expression levels of cytotoxic molecules on total natural killer (NK) cells in healthy controls (HCs) (n = 18), WARD (n = 34), ICU (n = 61) and post-COVID-19 patients (n = 28), upon return to the hospital six weeks after discharge. WARD (yellow) and ICU (red) samples were further subdivided based on the days post onset of COVID-19 symptoms. Pearson correlation between days post-symptoms and the indicated cytotoxic molecules in COVID-19 patients (bottom line). R = correlation coefficient. **(B)** Percentage expression of IFN-γ and TNF-α by total NK cells of indicated groups. **(C)** Contour plot showing IFN-γ and TNF-α expression by NK cells in HCs, WARD, and ICU COVID-19 patients. **(D)** Percentage expression of CD107a, CD69, CD49a, CD16, CD57, and HLA-DR on total NK cells. **(E)** Pearson correlation matrix for the tested markers. **(F)** Contour plot showing CD16 and CD49a expression on NK cells of indicated experimental groups. Each symbol represents a single patient. For the comparison of groups, *p*-values were obtained using Wilcoxon signed-rank test with Bonferroni correction. * Represent differences between indicated groups, ^#^ represent differences with HCs. */^#^p<0.05; **/^##^p<0.01; ***/^###^p<0.001; ^####^p<0.0001.

In order to kill target cells, NK cells must upregulate cytotoxic molecules which we investigated by intracellular flow cytometric analysis. We found a significantly increased expression (% and/or MFI) of perforin (PRF), granzyme A (GZMA), granzyme B (GZMB), and granzyme K (GZMK) in NK cells of COVID-19 WARD patients, whereas these were not different in ICU patients compared to HCs, except for GZMA that was decreased and GWMK that was increased (**Figure 3A** upper panels). When data are plotted according to the days of symptoms, we observed increased expression of PRF, GZMA and GZMB at early time points of COVID-19 disease (day 0-10 and day 11-20 post onset of symptoms) as compared to HCs, whereas decreased expression in samples taken at later time point of COVID-19 disease (day 21-30 and >30 days post onset of symptoms) (**Figure 3A** middle panels and **Supplementary Figure 4A**). PRF and GZMB expression was significantly decreased in post-COVID patients. The profile of these cytotoxic molecules was seen in both CD56^dim^ and CD56^bright^ NK cell subsets wherein, as expected, levels were highest in CD56^dim^ NK cells (**Supplementary Figures 4B, C**). GZMK showed a different expression pattern as its expression was predominantly increased in ICU patients (**Figure 3A**, upper panel), a finding that was mainly due to increased GZMK levels in CD56^dim^ NK cells (**Supplementary Figures 4B, C**). Even more strikingly, post-COVID-19 patients were seen to have increased GZMK expression compared to HCs, WARD, and ICU patients (**Figure 3A** upper panel).

To study if the differences in cytotoxic effector molecule expression in NK cells of WARD vs ICU patients is related to disease duration, we performed a Spearman correlation analysis. This revealed a negative correlation of PRF, GZMA, and GZMB expression (and a positive correlation for GZMK) with disease duration (**Figure 3A** lower panels, line graphs).

In addition to cytotoxic molecules, NK cells may also produce cytokines. To detect intracellular cytokine production by flow cytometry, NK cells require *in vitro* stimulation with either cytokines or target cells. However, considering the inflammatory nature of the disease, we sought to analyse direct IFN-γ and TNF-α production, without prior stimulation. Importantly, we found expression of IFN-γ and TNF-α in NK cells of most of COVID-19 patients, and some of the post-COVID-19 patients. The frequency of cytokine-producing NK cells as well as the levels of IFN-γ and TNF-α were significantly higher in ICU than in WARD patients (**Figure 3B**, MFI data in **Supplementary Figure 5A** upper panels). Levels of IFN-γ in NK cells significantly increased in samples taken at later time points post-onset of symptoms whereas levels of TNF-α remained high amongst the 3 groups of patients when sampling was done at >10 days post onset of COVID-19 symptoms (MFI and percentages in **Supplementary Figures 5A, B** lower panels). **Supplementary Figures 5C, D** shows that in addition to CD56^bright^ NK cells, CD56^dim^ subset are also important cytokine-producing cells in COVID-19 patients. Furthermore, as shown in a representative flow cytometric staining (**Figure 3C**), a large proportion of NK cells of ICU patients were double-positive for IFN-γ and TNF-α.

To investigate the correlation of cytotoxic moledules and cytokines with NK cell differentiation and activation, cells were stained with an additional antibody panel including CD107a, CD69, CD49a, CD16, CD57, and HLA-DR. The expression of the tested differentiation and activation markers on NK cells of HCs versus COVID-19 patients in different stages of their disease are shown in **Figure 3D** and **Supplementary Figures 5A, B** and the correlation of these markers with cytotoxic molecules or with IFN-γ and TNF-α are shown in **Figure 3E**. Expression of CD107a was significantly increased in NK cells from WARD patients, and even higher CD107a levels were noted in ICU patients (**Figure 3D**). Interestingly, both IFN-γ, TNF-α, and CD107a expression showed a positive correlation with the activation marker CD69 and the adhesion molecule CD49a (red box in **Figure 3E**), and negatively correlated with CD16, CD57, GZMA, and/or GZMB (green box in **Figure 3E**). **Figure 3F** plots a representative flow cytometric image of the CD16 and CD49a expression on NK cells of HCs, WARD, and ICU patients. It shows the appearance of a CD49a^+^ NK cell population in COVID patients and a general low expression of CD16 in NK cells from ICU patients. HLA-DR is upregulated on activated NK cells and HLA-DR+ cells are characterised by a more intensive cytokine-induced IFN-γ expression (37). In this study, HLA-DR expression was significantly upregulated in ICU and post-COVID-19 patients (**Figure 3D**). Note that HLA-DR was positively correlating with CD49a, CD69, and GZMK, whereas inversely with GZMA, CD16, CD57, and PRF (**Figure 3E**).

**Figure 4:**
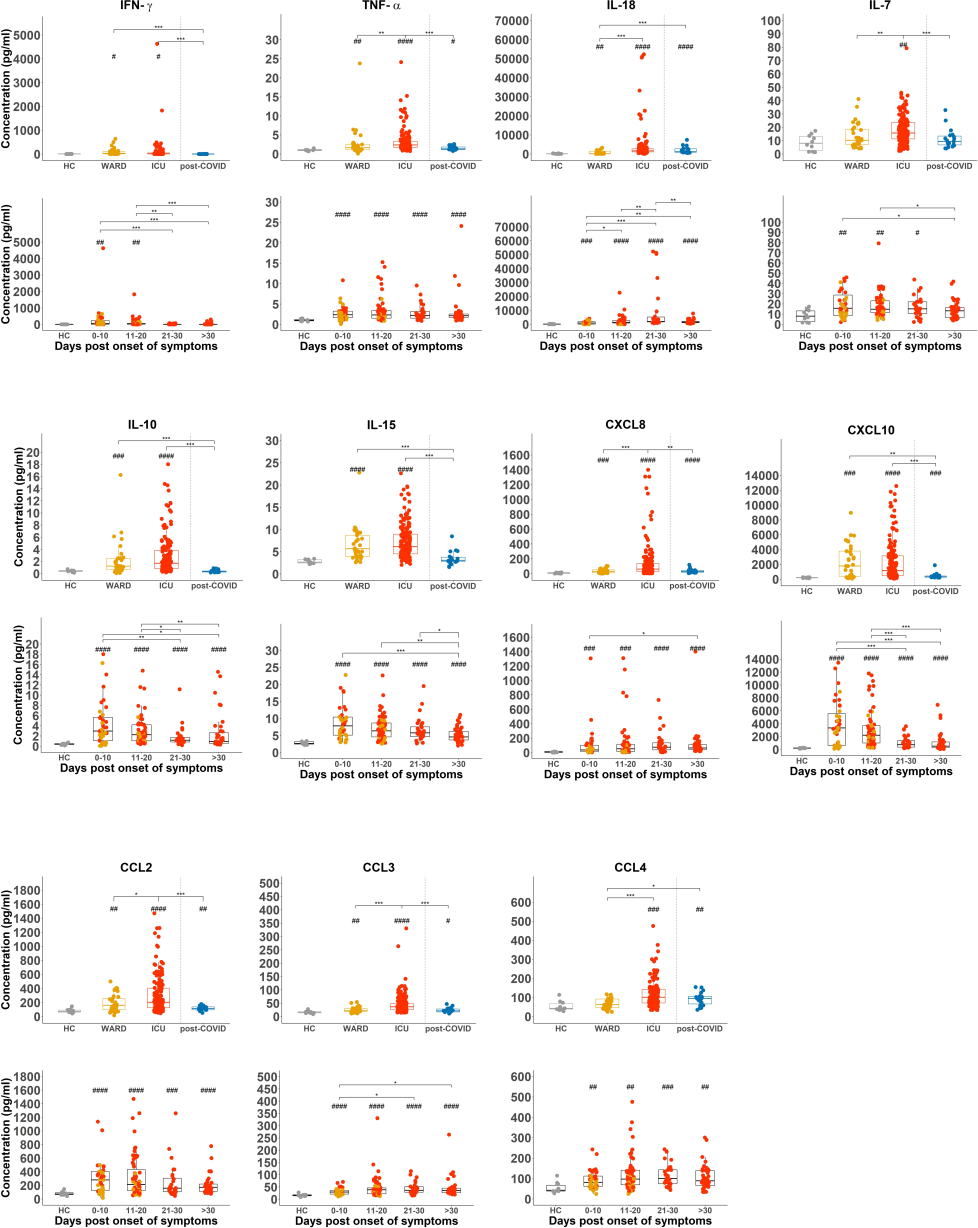
Increased NK cell-associated cytokines or chemokines and CXCL8 in in plasma of COVID-19 patients. Comparison of plasma levels of selected cytokines and chemokines from healthy controls (HCs) (n = 18), WARD (n = 34), ICU (n = 61), and post-COVID-19 patients (n = 28), upon return to the hospital six weeks after discharge. Plasma concentrations were measured by MSD (Meso Scale Discovery). Each symbol represents a single patient. For the comparison of groups, p-values were obtained using Wilcoxon signed-rank test with Bonferroni correction. * Represent differences between indicated groups, ^#^ represent differences with HCs. */^#^p<0.05; **/^##^p<0.01; ***/^###^p<0.001;****/^####^p<0.0001.

When plasma levels of cytokines and chemokines were analysed using a 30-plex kit, several of these (IFN-γ, TNF-α, IL-18, IL-7, IL-10, IL-15, CXCL8, CXCL10, CCL2, CCL3, and CCL4) were significantly increased in all COVID-19 patient groups as compared to HCs (**Figure 4**). Levels were most pronounced in samples taken at early time points post onset of COVID-19 symptoms with the exception of TNF-α and IL-18 which remained high in all hospitalised patients or even increased (IL-18) in samples taken at day 21-30 post symptoms (**Figure 4**). In post-COVID-19 patients, some plasma cytokines (i.e. TNF-α, IL-18, CXCL8, CXCL10, CCL2, CCL3, and CCL4) remained higher than in HCs, but levels were much lower as compared to hospitalised patients. Interestingly, cytokines and chemokines were found to correlate with CD69 expression on NK cells and to some extent to CD107a (**Supplementary Figure 6A**). All these cytokines and chemokines are associated with NK cell biology, either as an activating factor for – or as a product of – NK cells. Of special interest is the correlation of CD49a, CD69, and CD107a expression (corresponding with the cytokine-producing NK cells, **Figure 3E**) with plasma IL-15, a key cytokine in NK cell development and activation (38) that was previously linked to an attenuated NK cell inflammation signature in fatal COVID-19 (27). In our study, the emergence of this CD49a^+^CD69^+^CD107a^+^ NK cell subset was also correlated to the number of neutrophils, which is a surrogate disease severity marker (**Supplementary Figure 6B**) (35).

In summary, while NK cells from COVID-19 WARD patients have increased expression of cytotoxic molecules, NK cells from ICU patients have higher CD107a expression and have greatly increased production of cytokines in the absence of deliberated *in vitro* simulation. Throughout the progression of COVID-19, the cytotoxic effector molecules in NK cells significantly decrease whereas those of IFN-γ and TNF-α increase. While cytokine-producing NK cells are still present in the circulation of post-COVID-19 patients, their frequencies were decreased.

### NK cells of COVID-19 ICU patients display a defect in IL-12+IL-18 induced IFN-γ production

The elevated cytokine levels of IFN-γ and TNF-α in NK cells of COVID-19 patients as we found by our *ex vivo* analysis, contradict with the reported decreased cytokine production when cells of COVID-19 patients are stimulated *in vitro* with either cytokines or tumour target cells (31, 33). To understand this discrepancy, we stimulated PBMCs from part of our patient cohort for 18h with IL-12+IL-18, and subsequently analysed NK cell-produced IFN-γ and TNF-α by intracellular flow cytometry. As shown in **Supplementary Figure 7**, NK cells of COVID-19 ICU patients failed to produce IFN-γ upon stimulation with IL-12+IL-18 while those of WARD patients are diminished in their production as compared to the HCs. TNF-α levels in NK cells presented no differences between HCs and COVID-19 patients.

### Impaired *in vitro* NK cell cytolytic activity against tumour target cells in some severe COVID-19 patients

**Figure 5:**
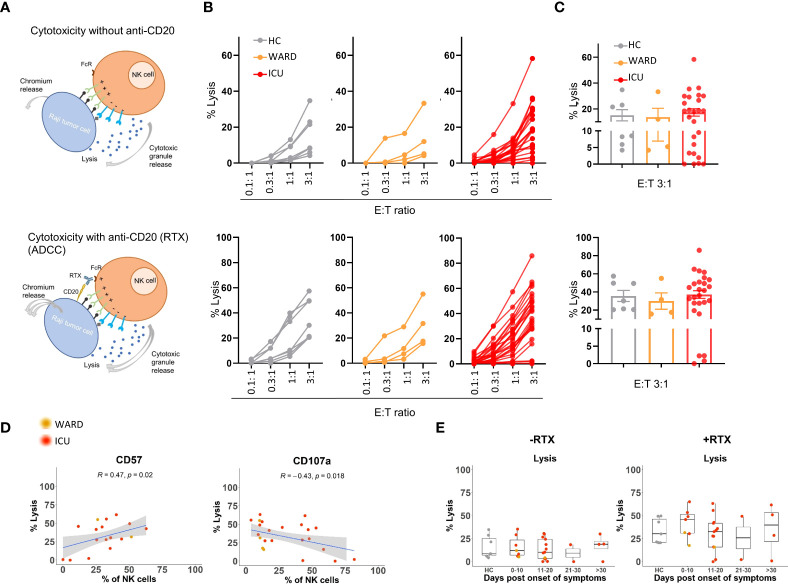
Direct and indirect (ADCC) cytolytic activities of NK cells from WARD and ICU patients. **(A)** Schematic overview of NK cell killing without or with anti-CD20 (Rituximab, RTX). Enriched NK cells were incubated for 4h with Chromium 51-labeled Raji cells and the cytolytic activity (% lysis) was calculated by the release of Chromium 51 as described in M&M. **(B)** Percentage lysis at indicated E:T ratio’s for healthy controls (HCs) (n = 7) (left, grey), WARD (n = 4) (middle, orange), and ICU (n = 26) (right, red) COVID-19 patients. **(C)** Representative bar graphs with the percentage of lysis for E:T 3:1 ratio are shown. Each symbol represents a single patient. **(D)** Pearson correlation between the percentage of lysis for E:T 3:1 ratio and the expression of CD57 and CD107a on NK cells of COVID-19 patients. R = correlation coefficient. **(E)** Data from panel C plotted after grouping of WARD and ICU patients according to the days post onset of COVID-19 symptoms. ADCC, Antibody-dependent cellular cytotoxicity.

To investigate the broader cytotoxic capacity of NK cells from COVID-19 patients against tumour target cells and their capacity to perform antibody cell-mediated cytotoxicity (ADCC), we performed ^51^chromium-release assays. We used Raji B cells (derived from Burkitt’s lymphoma) as NK cell targets because they express CD20, which provides a way to additionally analyse ADCC of NK cells by adding rituximab (i.e. anti-CD20 antibody) to the assay. Direct cytolysis of Raji cells by NK cells was measured in the absence of rituximab. The cytolytic assay is schematically presented in **Figure 5A** and was performed with NK cell-enriched samples from 4 WARD patients and 26 ICU patients. 7 HC samples were included. The percentage of target cell lysis was plotted for four different effector:target (E:T) ratios and averages for ratio 3:1 are depicted in a bar graphic (**Figures 5B, C**). NK cells from WARD and ICU patients were capable to kill Raji target cells equally well as HCs, both in the absence or presence of rituximab. NK cells from 7 out of 26 ICU patients were poorly cytolytic as seen by a low level of direct cell lysis. In the presence of rituximab, the complete defect in cytolysis remained in 4 of these 7 ICU patients.

To explain the weak cell-mediated cytotoxicity in these specific ICU patients, we examined whether their NK cells showed any phenotypic abnormalities. While we could not find a link with any of the NK cell-activating and inhibitory receptors, nor with the expression of PRF, GZMA, and GZMB, we noticed a positive correlation between target cell lysis and the expression of CD57 (R=0.47, p=0.02) by NK cells. Additionally, a negative correlation between target cell lysis and the expression of CD107a (R=-0.43, p=0.02) by NK cells was found (**Figure 5D**). Remark that the defective NK cells were not restricted to blood samples taken at the late phase of COVID-19 disease (**Figure 5E**).

Thus, while 4 ICU patients failed to kill target cells either directly or *via* ADCC, the majority of COVID-19 patients we analysed in this study displayed normal NK cell cytotoxic activity against Raji target cells.

### Transcriptional profile of 5 NK cell subsets in COVID-19 patients

We previously described heterogeneity in PBMCs isolated from COVID-19 WARD (n=14) and ICU (n=12) patients, based on scRNA-seq (36). NK cells formed a separate cluster, representing 7.9% and 7.4% of total PBMCs in WARD and ICU patients respectively, and were identified by expression of marker genes *NCR1* (NKp46), *NCAM1* (CD56), *KLRB1* (CD161), *NCR3* (NKp30), *KLRD1* (CD94), *KLRF1* (NKp80), *FCGR3A* (CD16), natural killer cell granule protein 7 (*NKG7*) and the TYRO binding protein (*TYROBP*) (36). Here, we additionally dissected the NK cells, exploring NK cell heterogeneity. To compare NK cells from COVID patients with HCs, we merged our single-cell data with the PBMC dataset from Wilk et al. (17), adding 6 HCs, 4 WARD, and 4 ICU patients (using similar clinical criteria as in our cohort).

**Figure 6:**
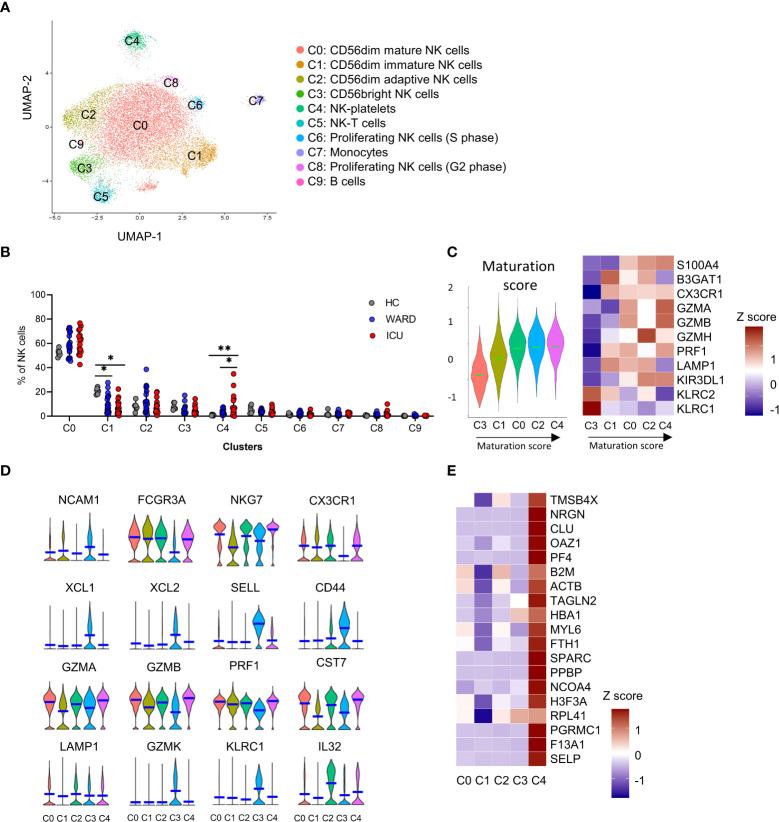
Single-cell RNA-sequencing reveals 10 distinct NK cell subsets. **(A)** UMAP representation of 16560 sub-clustered natural killer (NK) cells [obtained from peripheral blood mononuclear cells (PBMCs) from healthy controls (HCs) (n = 6), WARD patients (n = 16) and ICU patients (n = 16)] by scRNA-seq, colour-coded for the indicated cell type. **(B)** The relative contribution of each NK cell type (in %) in HCs, WARD, and ICU COVID-19 patients. **(C)** Heatmap of maturation-related genes and representative score in the different clusters. **(D)** Violin plots of key NK cells marker genes in the different subclusters. **(E)** Heatmap of main platelet-related genes. P-values were obtained using Wilcoxon signed-rank test with Bonferroni correction. *p<0.05; **p<0.01.

In our analysis, unsupervised clustering identified 10 subclusters (C0 – C9; **Figures 6A, B** and **Supplementary Figure 8**). These comprised of CD56^dim^ mature NK cells (C0), CD56^dim^ immature NK cells (C1), CD56^dim^ adaptive NK cells (C2), CD56^bright^ NK cells (C3), and NK-platelets (C4). The other remaining clusters (C5-9) were defined as NK-T cells (C5), proliferating NK cells (C6 and C8), monocytes (C7), and B cells (C9) and are described in the **Supplementary M&M**. Cells from the two merged datasets (**Supplementary Figure 9A**) and cells from the different patients and HCs clustered together (individual patient stratification is shown in **Supplementary Figure 9B**).

The maturational state of the different NK cell-related clusters (C0-4) is presented in **Figure 6C**. C3 NK cells were the least mature and were annotated as CD56^bright^ NK cells based on the expression of *NCAM1* (CD56), *XCL1*, *XCL2*, *SELL* (CD62L), *CD44*, granzyme K (*GZMK*), high expression of *KLRC1* (NKG2A), and low expression of *FCGR3A* (CD16) (**Figure 6D**). A comparison with the gene expression published by Collins et al. (39) further supported the identity of the CD56^bright^ NK cells (**Figure 6D** and **Supplementary Figure 10A**). C3 cells represented 7.9% of the NK cells in HCs and were decreased in WARD (5.4%) and ICU (4.7%) patients, albeit non-significantly (**Figure 6B**).

Based on the maturation score, we noticed a gradual maturation from the CD56^bright^ NK cells (C3), into CD56^dim^ mature NK cells (C0) and CD56^dim^ adaptive NK cells (C2) *via* a CD56^dim^ immature NK cell subcluster (C1). The NK-platelets (C4) mainly showed a mature phenotype. We used pseudotime trajectory to confirm the differentiation trajectory of the 4 main NK cells populations (C0-C3) in which we defined C3 CD56^bright^ NK cells as the root of the trajectory (**Supplementary Figure 9C**).

The C0 CD56^dim^ mature NK cells (**Figure 6C**) display a low expression of *NCAM1* (CD56), a high expression of lytic granule genes such as perforin (*PRF1*), granzyme A and B (*GZMA*, *GZMB*), and a high expression of the cytolytic regulator *CST7*, which is needed for optimal cytotoxicity (40) (**Figure 6D**). The percentage of C0 NK cells was non-significantly increased in WARD and ICU patients (58.8% and 60.8% respectively) compared to HCs (52.3%). Compared to C0, C1 mainly showed a lower expression of cytotoxic markers including GZMA, GZMB, GZMH, and S100A4 (**Figure 6C**). The C1 subset represented 20.8% of the total NK cells in HCs and was significantly decreased in WARD and ICU patients respectively (10.8% and 5.0% of the total NK cells respectively), demonstrating a shift towards functionally more mature and active NK cells (**Figure 6B**).

Maucourant et al. described adaptive NK cells in COVID-19 patients (26). In our study, cluster C2 represents CD56^dim^ cells with an adaptive NK cell phenotype. This was based on the gene signature described by Schlums et al. (41), showing low expression of *KLRC1* (NKG2A), *ZBTB16* (PLZF), *FCER1G* (FCϵR1γ), and high expression of *KLRC2/3/4* (NKG2C/E/F), Granzyme H (*GZMH*), *CCL5*, *CD3E*, and *IL-32* (**Figure 6D** and **Supplementary Figure 10B**). In HCs, 8.0% of all NK cells were annotated as adaptive NK cells, and percentages increased in WARD patients (13.0%), albeit non-significantly. The percentage of C2 subset NK cells was unaltered in ICU patients (7.9%) (**Figure 6B**).

Intriguingly, C4 NK cells were referred to as an NK cell-platelet subset since these cells expressed platelet-related genes *PPBP*, *CLU*, *F13A1*, *NRGN*, *OAZ1*, *PF4*, *SELP*, *SPARC, TAGLN2*, amongst others, along with NK cell markers (**Figures 6D, E**, and **Supplementary Figure 8**). The NK cell-platelet subset was COVID-19-specific since they were barely present in HCs (0.9%) but significantly increased in WARD (3.5%) and ICU patients (on average 12.1% and with individual percentages up to 34.9%) (**Figure 6B**).

**Supplementary Figure 11** describes the activational state of the different NK cell subsets in HCs, WARD and ICU COVID-19 patients. Based on the expression of cytotoxic molecules and cytokines, increased activation was seen in patients (**Supplementary Figures 11A, B**). Interestingly, in agreement with our flow-cytometric analysis, the cytokine expression in both the CD56^dim^ and CD56^bright^ NK cells was higher in patients compared HCs. (**Supplementary Figure 11B**). Of note, no exhausted phenotype was found since exhaustion markers were rather decreased in patients when compared to HCs (**Supplementary Figure 11C**).

Taken together, SARS-CoV-2 infection triggers a redistribution of NK cell subsets towards a more mature and activated phenotype. Unexpectedly severe COVID was associated with the emergence of a – so far undescribed – NK cell-platelet subset.

### Blood platelets adhere to NK cells of COVID-19 ICU patients

**Figure 7:**
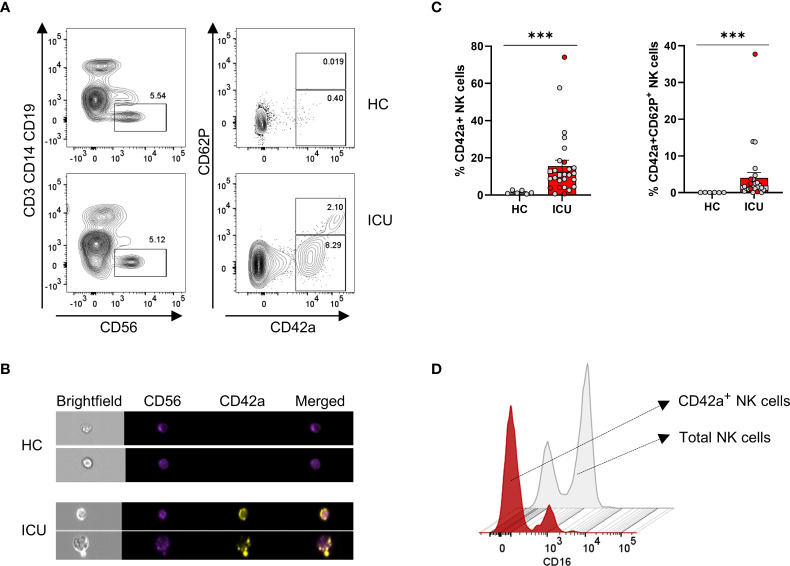
ICU patients present with platelet-NK cell aggregates in which platelets are largely activated. **(A)** Contour representation of platelet-NK cell aggregates with NK cells gated as CD3-CD14-CD19-CD56+ and platelets gated as CD42a+. Activated platelets are defined as CD42a+CD62P+. The top line depicts a representative healthy control (HCs), and the bottom line, a representative ICU COVID-19 patient. **(B)** Imaging flow cytometric visualisation of platelet-NK cell aggregates in two representative HCs and ICU COVID-19 patients. CD56 is visualised in red, CD42a in yellow, and a merged image in orange. **(C)** Percentage expression of CD42a+ and CD42a+CD62P+ NK cells in HCs (grey bar) (n = 6) and ICU COVID-19 patients (red bar) (n = 27). Each symbol represents a single patient. Coloured symbols were obtained from fresh samples. Mann-Whitney U test was used to determine the significance between HCs and ICU. **(D)** Mean fluorescence intensity (MFI) plot of CD16 on total NK cells in grey and platelet-NK cell aggregates in red. P-values were obtained using Wilcoxon signed-rank test with Bonferroni correction. ***p<0.001.

Our single-cell transcriptome analysis revealed a COVID-19 specific cell population in ICU patients with a gene signature of both NK cells and platelets (i.e. cluster 4, **Figures 6B, D, E**). We hypothesised that this subset results from platelets strongly adhering to NK cells and therefore performed flow cytometry on PBMCs that were stained both for NK cell markers (CD56 and NKp46) and markers of platelets (CD42a and CD62P, which are expressed on the surface of all platelets and activated platelets, respectively) (42, 43). As shown in a representative dot plot in **Figure 7A**, a fraction of NK cells from ICU patients stained positive for the CD42a platelet marker and part of these also expressed externalised CD62P, indicating degranulation by activated platelets. By imaging cytometry, we confirmed the adherence of platelets on NK cells of ICU patients. These NK cell-platelet complexes were seen in all investigated ICU patients (n= 18) but not in HCs (**Figures 7B, C**). While on average 12% of total NK cells from ICU patients were covered with platelets, these levels increased in some patients to more than 40%. NK-platelet aggregates showed reduced CD16 expression (**Figure 7D**). NK cell-platelet aggregates were not a freezing artifact as these were also depicted in freshly-isolated samples (coloured data points in **Figure 7C**). We found no correlations between the frequency of NK-platelet aggregates and the amount of cytotoxicity, which may be due to the small number of samples on which both assays were performed (n = 16).

These data clearly show that COVID-19 patients are represented with an NK cell-platelet cluster that is partially activated and is absent in HCs. Furthermore, NK-platelet doublets have decreased CD16 expression.

## Discussion

Consistent with other reports, we found that SARS-CoV-2 infection triggers maturation of NK cells leading to increased frequencies of CD56^dim^ NK cells (26, 29, 32, 44). The increased levels of terminally differentiated inflammatory NK cells in severe COVID-19 patients were also confirmed in two additional scRNA-seq datasets (33). Adaptive NK cells are another CD56^dim^ subset of terminally differentiated NK cells, were non-significantly increased in our COVID-19 cohort subjected to transcriptome analysis. Using flow cytometry, two studies found an increased frequency of these adaptive NK cells, phenotyped by either CD56^dim^NKG2C^+^CD57^+^ or FcεRig^-^CD56+CD57+ cells, in severe but not in moderate COVID-19 patients (26, 29). In contrast, Leem et al. and Witkowski et al., reported no significant numerical changes of adaptive NK cells (32, 33). In our flow cytometric antibody panel, we did not include NKG2C or other markers for adaptive NK cells. Whereas proliferating NK cells were strongly overrepresented in other studies (33), we only noticed a non-significant increased number of proliferating NK cells in COVID-19 patients.

Alongside the maturation of NK cells, SARS-CoV-2 infection also leads to robust activation of NK cells, as evident from the increased expression and protein levels of intracellular cytotoxic molecules and cytokines. In terms of cytotoxic effector molecules, there are substantial differences between WARD patients with mild or moderate disease symptoms and ICU patients with severe disease symptoms. Whereas NK cells from COVID-19 WARD patients show an increase in PRF, GZMA, and GZMB, levels were decreased in ICU patients. Plotting the cytotoxic levels of all hospitalised patients according to days post onset of COVID-19 symptoms shows that the differences in PRF, GZMA, GZMB, and GZMK between ICU and WARD patients are ascribed to differences in their disease stage, with a clear increase during the first 10 days of COVID-19 symptoms, followed by a decrease over the course of the disease. The cytokine production, as measured *ex vivo* by intracellular flow cytometry, follows an opposite pattern. NK cells from ICU patients expressed highest levels of cytoplasmic IFN-γ and TNF-α proteins. While IFN-γ levels in NK cells significantly increased during progression of COVID-19 disease, levels of TNF-α remained high in all samples taken >11 days post onset of COVID-19 symptoms. IFN-γ and TNF-α are well-described to synergise in the induction of ICAM1, a cell adhesion molecule on endothelial cells (45), and may explain the high extravasation of leukocytes to the site of inflammation (46). Remarkably, NK cell cytokine production and plasma cytokine levels remained high in post-COVID patients. We would like to emphasise that the expression of cytoplasmic presence of IFN-γ and TNF-α proteins was measured without *in vitro* stimulation with cytokines or target cells, which is usually required to detect cytokine proteins in NK cells. Other research groups reporting reduced cytokine expression in NK cells of COVID-19 patients (26–28, 30) have stimulated the cells *in vitro* with K562 target cells, PMA/ionomycin, or cytokines. In an additional experiment in which NK cells were stimulated *in vitro* for 18 hours with IL-12+IL-18, we confirmed the defective cytokine-induced IFN-γ production in patients with severe COVID-19 disease symptoms. The different outcome by using these two procedures may indicate that the NK cells in patients may have reached a state of saturation in terms of cytokine production. A defect in IL-18-induced IFN-γ by NK cells has been described in patients with systemic juvenile idiopathic arthritis (sJIA), an autoinflammatory rheumatological disease that is associated with development of MAS (47, 48). In these patients, the defect in IL-18-induced IFN-γ production has been linked to a defect in the phosphorylation of the IL-18 receptor most likely as a consequence of NK cell saturation by their high and prolonged IL-18 blood levels. High plasma levels of IL-18 are seen in our COVID-19 patient cohort (**Figure 4**) and distinguish severely ill from mild disease (36).

The presence of cytoplasmic IFN-γ and TNF-α proteins, measured *ex vivo*, highly correlated with cell membrane expression of CD49a, CD69, and CD107a, raising the question whether these CD69^+^CD49a^+^CD107a^+^ cytokine-producing NK cells in severe COVID-19 patients represent a distinct subset of NK cells. In this respect, it is noteworthy to mention that CD69, as well as CD49a, are two markers of trNK cells, also designated in mice as type I innate lymphoid cells (ILC1) (49, 50). trNK home in non-lymphoid organs but in severe inflammatory diseases, such as sepsis, they may lose their residence and move into the circulation (51). trNK cells are well known to display lower cytotoxic activity and a higher cytokine production than conventional NK cells (52, 53). The CD69^+^CD49a^+^CD107a^+^ cytokine-producing NK cells defined in our study may correspond with the increase in CD69^+^CD103^+^CXCR6^+^ NK cells described in COVID-19 patients by Bozzano et al. (30). Indeed, in addition to CD69 and CD49a, CD103 and CXCR6 are markers of trNK cells and are functionally involved in tissue retention (50). Given that the CD49a^+^ NK cell population in our study barely expresses CD16 (**Figure 3F**), our cytokine-producing NK cell subset may also correspond to the CD56^dim^CD16^neg^ NK cell population described by Leem et al. in COVID-19 patients with severe disease (32). It can be noted that if the cytokine-producing NK cell subset in our study represents trNK cells, one would expect these cells to appear as a distinct cluster in our scRNA-seq. However, ICU samples for the transcriptome analysis were taken on admission (with one exception) and our flow cytometric data revealed that the phenotypic switch only occurs during a longer stay at ICU. Taken together, the emergence of a distinct population of NK cells in COVID-19 patients with severe disease is a point of major interest. The origin of these cells as well as their function has yet to be determined. Given their potent ability to produce high levels of cytokines and chemokines, it will be of interest to explore whether these cells contribute to immunopathology and organ damage.

Concerning the broader cytolytic activities of NK cells, we demonstrated that NK cells from the majority of COVID-19 patients kill Raji tumour cells *in vitro* equally well as those from HCs. However, NK cells from 4 out of 26 ICU patients (15%) completely failed to kill tumour target cells, both in an ADCC-dependent and independent assay. A clear-cut explanation for the complete failure of NK cell cytolytic activity in the 4 ICU patients was not found, as the cells showed comparable levels of perforin and granzymes, comparable to those from COVID-19 patients with normal killing capacity. Recently, a decreased cytotoxicity in NK cells from severe COVID-19 patients was linked to either an exaggerated IFN-α or TGF-β response (33). The reduced cytotoxicity of NK cells as reported in COVID-19 patients is often concluded from decreased levels of perforin and granzymes or from decreased degranulation as analysed by CD107a expression after stimulation with tumour target cells (19, 28–30). Three studies directly demonstrate an impaired cytolytic killing activity of NK cells from severe COVID-19 patients (31–33), and there are a number of reasons that may explain why cytolytic defects were not detected in the majority of our patients. We opted to use Raji cells in order to combine with an ADCC assay, and these cell line is known to be a less sensitive NK cell target as compared to K562 cells. Incomplete cytotoxic defects in our study might also be due to a lower effector-target ratio used, a different time point on which samples were obtained (as the reported impaired killing was mainly seen within the first week of diagnosis (32)), or a different pre-incubation stimulus (i.e. IL-15 alone in our study compared to combined IL-15 + IL-12 pre-stimulation). From this, it can be concluded that only part of the severe COVID-19 patients had intrinsic or persistently acquired cytolytic defects.

Our scRNA-seq revealed in COVID-19 patients a distinct NK cell population with common genes specific for blood platelets. By flow cytometry and imaging flow cytometry we validated this subset and identified platelet-NK cell aggregates in COVID-19 ICU patients. The aggregates were phenotyped by CD42a-positivity on NK cell events. CD42a, also known as glycoprotein IX that is constitutively expressed by platelets as part of the GP1b-V-IX complex on the platelet surface (54). This glycoprotein is not cleaved by sheddases including ADAM17 upon platelet activation (55) and thus represents a stable marker for platelets. A subset of the aggregates also expressed CD62P (also known as P-selectin), a binding partner of PSGL-1 which is present in platelet α-granules and ospitalised to the cell surface upon platelet activation (56). It has been shown that platelets interact with monocytes, neutrophils, and T cells to form aggregates (57), but to our knowledge, platelet-NK cell aggregates have not been described. In COVID-19 patients circulating platelet-neutrophil, platelet-monocyte aggregates, and platelet-lymphocyte aggregates have been reported (58–61). A common feature of these aggregates is that in patients with severe disease, both cell types (leukocytes and platelets) present a hyperactivated state. In the study by Hottz et al., platelet-monocyte aggregates were associated with increased activation markers on platelets and increased expression of tissue factor by the monocytes. Platelet activation and tissue factor expression were associated with markers of coagulation exacerbation i.e. fibrinogen and D-dimers, leading the authors to conclude that these aggregates exacerbate the coagulopathy of COVID-19. Similarly, as described by Middleton et al. (59) and others (62), platelet-neutrophil complexes lead to the release of neutrophil extracellular traps which further exacerbate COVID-19 coagulopathy. Concerning the platelet-NK cell aggregates that we found in COVID-19, we speculate on a similar scenario that both cell types are hyperactivated and contribute to the disease pathology. Considering that activated platelets and NK cells are potent producers of cytokines and chemokines, the aggregates may exacerbate the cytokine storm syndrome. The hyperactivated status of platelets is well documented in COVID patients and is evidenced by the increase in various of their activities, such as platelet adhesion and aggregation, antigen presentation and production of ROS and cytokines (60, 63–65). While the platelets can be activated indirectly by soluble factors (a likely candidate is complement C5a (65), it is also possible that they are directly responding to SARS-CoV-2, as specific viral RNA molecules have been found in platelets of at least some of COVID-19 patients (60, 63). The effects of activated platelets on the cytolytic activity of NK cells are difficult to predict. In our experiments, we found decreased expression of CD16 on the NK cell-platelet aggregates (as compared to non-platelet-bound NK cells) which may be explained by the activity of ADAM17, a metalloproteinase that cleaves CD16 and that is expressed by activated platelets and NK cells (55, 66, 67). Given their low CD16 expression – required for proper ADCC – in our experiments, we found no significant correlation between NK-mediated tumour cell lysis and the frequency of platelet-NK cell aggregates.

While the role of NK cell platelet aggregates in viral infections is a topic requiring further investigation, another interesting research question is which ligands and receptors are involved in the NK cell platelet interaction. Here one can speculate about some mechanisms as described for platelet-monocyte/neutrophil aggregates (68). A very likely candidate in the formation of NK cell platelet aggregates is P-selectin, which is externalised by activated platelets and which interacts with P-selectin glycoprotein ligand (PSGL-1), expressed by resting and activated NK cells (69).

The low number of NK cells in post-COVID-19 patients, who recovered from the disease and who had been discharged from the hospital six weeks earlier, was noteworthy. Lymphopenia and its slow repair are well known in COVID-19 patients who have been hospitalised (70), nevertheless, it was still a surprise to find NK cell counts to be lower than in all other groups of our study cohort. The remaining NK cells in these patients exhibited an activated phenotype that, together with the elevated pro-inflammatory cytokines in plasma, is consistent with the attenuated medical and physical condition of the patients. The data argue for longer monitoring of post-COVID-19 patients.

Our study has some limitations. First, the healthy donors were not age-matched as their median age was significantly lower than those of COVID-19 patients (39 years in HCs versus 60 years and 64 years in ICU and WARD respectively, **Supplementary Table 1**). Studies described that older people have a decreased proportion of CD56^bright^ versus CD56^dim^ NK cells and a decreased capacity to produce cytokines, chemokines, and cytotoxic molecules (71, 72). Therefore, in the present study, the numerical differences in NK cells between patients and HCs, but not their cytotoxic and cytokine-producing activities, may be attributed to age differences. However, given that the mean ages of WARD, ICU, and post-COVID-19 patients do not differ, the key findings described in our study cannot be ascribed to age differences. Second, NK cell analysis was done on PBMCs which may not reflect the process of inflammation in tissues. Concentrations of proteases and cytokines are likely increased in lung tissue of COVID-19 patients as compared to levels in blood (73), and these may change the expression of NK cell receptors (such as CD16) and may drive NK cell activities into an exhausted state. Third, our study did not include a cohort of patients with other lung infections, for example influenza virus, so we cannot infer whether our findings are COVID-19-specific or more generally reflect severe lung infections. Consistent with our findings, hyperactivation of NK cells in terms of increased CD107a degranulation and production of IFN-γ and TNF-α has been described in the blood of patients during acute influenza A infection (74). Intriguingly, NK cell hyperactivation during influenza A infection was most evident in a population of CD56^+^CD16^-^CD49a^+^CXCR3^+^ NK cells (74), thus corresponding to the cytokine-producing subset of our study. The authors described the population as trNK cells and in line with our thoughts postulate that the cells contribute to disease pathology.

Taken together, our study demonstrates that NK cells participate in the innate immune response of SARS-CoV-2 infection. The cells are highly activated in terms of their cytotoxic and cytokine-producing aspects. The clear-cut and inversely correlated differences in the phenotype of cytotoxic NK cells in WARD patients with mild disease to cytokine-producing NK cells in ICU patients with severe disease are indicative for either a cytokine-driven skewing or the appearance in the circulation of a CD49a^+^CD69a^+^CD107a^+^ NK cell subset which may represent trNK cells. While the lower amounts of cytotoxic molecules in NK cells of patients with severe symptoms are suggestive of impaired cytotoxic activity, the majority of COVID-19 NK cells displayed a normal cytotoxic killing of Raji tumour target cells. Another point of interest is the presence of platelet-NK cell aggregates in patients with severe symptoms that may exacerbate the cytokine storm and coagulopathy of severe COVID-19. The low NK cell count with a cytokine-producing phenotype in patients recovering from COVID-19 indicates that post-COVID patients have a slow NK cell recovery that may compromise the innate immune system, confirming the need for monitoring patients after hospital discharge. From a fundamental perspective, it will be important to explore whether and how trNK cells and platelet-NK aggregates contribute to antiviral immunity and pathology.

## Additional CONTAGIOUS collaborators

Francesca Bosisio, Christophe Dooms, Natalie Lorent, Sirima Kraisin, Johan Neyts, Sabine Tejpar, Karin Thevissen, Thomas Tousseyn, Birgit Weynand, Toine Mercier, Erwin Dreesen, Jonas Yserbyt, Dries Testelmans.

## Data availability statement

Publicly available datasets were analysed in this study. This data can be found here: EGA European Genome-Phenome Archive database (EGAS00001004717) and the gene expression omnibus (GSE145926).

## Ethics statement

The studies involving human participants were reviewed and approved by UZ Leuven Ethical Committee. The patients/participants provided their written informed consent to participate in this study.

## Author contributions

BM-D, JF, KA, BB, JV, AG, SH-B, FDS, KM, EW, PP, CW, GL, DL, JW, and PM designed the experiments, developed the methodology, analysed and interpreted data, and wrote the manuscript. LV, PVM, YVH, GH, PMe, AW, EW, CW, and JW performed sample collection. BM-D, JF, KA, BB, JV, ADV, EB, TM, and MG performed experiments and acquired data. CJ, LV, PVM, GH, YVH, PMe and AW provided technical support and supported data analysis and interpretation. DL, JW, and PM supervised the study and were responsible for coordination and strategy. BM-D and PM were responsible for the final assembly and submission of the manuscript. All authors have approved the final manuscript for publication.

## Funding

This work was funded by the Research Foundation – Flanders (FWO-Vlaanderen) (G.0A32.18N), a C1 grant from KU Leuven (C16/17/010), a UZ-Leuven/KOOR grant for COVID-19 research (ESACOVID2-O2010), the Rega foundation (research expert fellowship to MG). PM received support from the European Union’s Horizon 2020 research and innovative programme under grant agreement No. 779295. KA and JV received an FWO-SB fellowship for strategic research 1S10618N and 1S75320N respectively. LV and PVM are supported by an FWO PhD fellowship (grant numbers 11E9819N, 1S66020N). EW is supported by Stichting tegen Kanker (Mandate for basic & clinical oncology research). JW is supported by an FWO Fundamental Clinical Mandate (1833317N).

## Acknowledgments

We thank prof. Stefaan Soenen, Christy Maksoudian and Carla Rios Luci (department of imaging and pathology, KU Leuven) for the use of the Imagestream.

## Conflict of interest

The authors declare that the research was conducted in the absence of any commercial or financial relationships that could be construed as a potential conflict of interest.

## Publisher’s note

All claims expressed in this article are solely those of the authors and do not necessarily represent those of their affiliated organizations, or those of the publisher, the editors and the reviewers. Any product that may be evaluated in this article, or claim that may be made by its manufacturer, is not guaranteed or endorsed by the publisher.
