# Supplementary material for: Severe COVID-19 patients display hyper-activated NK cells and NK cell-platelet aggregates
Source: Front Immunol. 2022 Oct 5;13:861251. doi: 10.3389/fimmu.2022.861251 (PMC9581751; doi:10.3389/fimmu.2022.861251)
Supplement: Supplementary file 1 [file DataSheet_1.pdf]

Severe COVID-19 patients display hyper-activated NK cells and NK cell-platelet aggregates

Bert Malengier-Devlies[1*], Jessica Filtjens[1*], Kourosh Ahmadzadeh[1], Bram Boeckx[2], Jessica Vandenhaute[1], Amber De Visscher[1], Eline Bernaerts[1], Tania Mitera[1], Cato Jacobs[3], Lore Vanderbeke[4], Pierre Van Mol[2], Yannick Van Herck[5], Greet Hermans[6], Philippe Meersseman[3], Alexander Wilmer[3], the CONTAGIOUS consortium, Mieke Gouwy[7], Abhishek D Garg[8], Stephanie Humblet-Baron[9], Frederik De Smet[10], Kimberly Martinod[11], Els Wauters[12], Paul Proost[7], Carine Wouters[1] , Georges Leclercq[13], Diether Lambrechts[2*], Joost Wauters[3*] and Patrick Matthys[1*].

[1] Laboratory of Immunobiology, Department of Microbiology, Immunology and Transplantation, Rega Institute, KU Leuven, Leuven, Belgium
[2] Laboratory of Translational Genetics, Department of Human Genetics, VIB-KU Leuven, Leuven, Belgium
[3] Laboratory for Clinical Infectious and Inflammatory Disorders, Department of Microbiology, Immunology and Transplantation, KU Leuven, Leuven, Belgium
[4] Laboratory of Clinical Bacteriology and Mycology, Department of Microbiology, Immunology and Transplantation, KU Leuven, Leuven, 3000, Belgium
[5] Laboratory of Experimental Oncology, Department of Oncology, KU Leuven, Leuven, 3000, Belgium
[6] Laboratory of Intensive Care Medicine, Department of Cellular and Molecular Medicine, KU Leuven, Leuven, 3000, Belgium
[7] Laboratory of Molecular Immunology, Department of Microbiology, Immunology and Transplantation, Rega Institute, KU Leuven, Leuven, Belgium
[8] Laboratory for Cell Stress & Immunity (CSI), Department of Cellular and Molecular Medicine (CMM), KU Leuven, Leuven, 3000, Belgium
[9] Adaptive Immunology, Department of Microbiology, Immunology and Transplantation, KU Leuven, Leuven, Belgium
[10] Laboratory for Precision Cancer Medicine, Translational Cell and Tissue Research, Department of Imaging & Pathology, KU Leuven, Leuven, 3000, Belgium
[11] Centre for Molecular and Vascular Biology, Department of Cardiovascular Sciences, KU Leuven, Leuven, Belgium
[12] Laboratory of Respiratory Diseases and Thoracic Surgery (BREATHE), Department of Chronic Diseases and Metabolism, KU Leuven, Leuven, 3000, Belgium
[13] Laboratory of Experimental Immunology, Department of Diagnostic Sciences, Ghent University, Ghent, Belgium

*Equal contribution as first or last author

Supplemental material

Supplementary Tables

Supplementary Table 1. Patient demographics and disease characteristics

	Total (n)	M (%)	Measurements (#)	Age (years)	WBCs (10^9/L)	Neutrophils (10^9/L)	Lymphocytes (10^9/L)	Monocytes (10^9/L)	Platelets (10^9/L)	CRP (mg/L)	AST (U/L)	ALT (U/L)	Ferritin (μg/L)	D-dimers (μg/L)
HC	18	55.6	18	39 (25-65)	NA	NA	NA	NA	NA	NA	NA	NA	NA	NA
ICU	61	80.0	164	60 (37-85)	9.09 (0.77-52.5)	6.7 (0.4-38.3)	1.2 (0.1-5.2)	0.6 (0-32.3)	332 (27-835)	80 (1-474)	44 (15-680)	43 (5-876)	1262 (79-85086)	1749.5 (314-42785)
WARD	34	55.8	34	64 (23-91)	6.68 (1.79-16.37)	4.5 (1.1-13.3)	1.2 (0.4-3.2)	0.6 (0.2-1.1)	243 (75-533)	58 (0.3-272)	28 (12-913)	25 (8-861)	558 (23-2496)	916 (215-4128)
Post-COVID	28	67.9	28	57 (23-75)	7.19 (3.72-26.25)	4.4 (1.6-10.3)	2.2 (0.9-5.1)	0.5 (0.3-1.0)	284 (182-431)	4 (0.3-18)	22 (13-77)	18 (8-110)	175 (35-819)	439 (215-1100)
Post-ICU	20	80.0	20	57 (42-75)	7.22 (5.52-10.26)	4.5 (3.2-6.6)	2.2 (0.9-3.5)	0.5 (0.3-0.9)	293.5 (182-431)	4 (0.3-18)	22 (14-77)	19 (11-110)	178 (35-819)	446 (215-1100)
Post-WARD	8	37.5	8	57 (23-69)	6.82 (3.72-16.59)	4.1 (1.6-10.3)	2.4 (1.7-5.1)	0.4 (0.3-1)	269 (212-363)	1 (0.3-6)	19 (13-28)	16 (8-33)	89 (44-594)	311 (229-595)

Data are median (Range). CRP: C-reactive protein; ALT: alanine aminotransferase; AST: aspartate aminotransferase; NA: no data acquired.

Supplementary Table 2. Comorbidities of COVID-19 patients

	**Ward (n=36)**	**ICU (n=61)**	**Total (n=97)**
History of diabetes	7 (19%)	13 (21%)	20 (21%)
Prior myocardial infarction	2 (6%)	2/59 (3%)	4/95 (4%)
Congestive heart failure	3 (8%)	2 (3%)	5 (5%)
Arterial hypertension	22 (61%)	32 (52%)	54 (56%)
Peripheral vascular disease	1 (3%)	2/60 (3%)	3/96 (3%)
Cerebrovascular disease	1 (3%)	1/60 (2%)	2/96 (2%)
Chronic pulmonary disease	3 (8%)	8 (13%)	11 (11%)
Asthma	2 (6%)	1 (2%)	3 (3%)
COPD	1 (3%)	5 (8%)	6 (6%)
ILD	0	2 (3%)	2 (2%)
Smoking history			
Active	*1 (3%)*	*5 (8%)*	*6 (6%)*
Former	*12 (33%)*	*19 (31%)*	*31 (32%)*
Never	*13 (36%)*	*24 (39%)*	*37 (38%)*
Unknown	*10 (28%)*	*13 (22%)*	*23 (24%)*
Rheumatological disease	2 (6%)	8/59 (14%)	10/95 (11%)
Peptic ulcer disease	2 (6%)	2/60 (3%)	4/96 (4%)
Underlying liver disease	1/35 (3%)	0/59	1/94 (1%)
Moderate-to-severe renal disease	0	1/60 (2%)	1/96 (1%)
Chronic dialysis	-	*1 (2%)*	*1 (1%)*
Active hematologic malignancy	0	1 (2%)	1 (1%)
Active solid cancer	3 (8%)	4 (7%)	7 (10%)
Cervix carcinoma	*1 (3%)*	*0*	*1 (1%)*
DLBCL	*0*	*1 (2%)*	*1 (1%)*
Prostate carcinoma	*2 (6%)*	*1 (2%)*	*3 (3%)*
Sigmoid adenocarcinoma	*0*	*1 (2%)*	*1 (1%)*
History of cancer	3 (8%)	5/58 (9%)	8/94 (9%)
HIV	0/9	0/35	0/44
Median LDH in U/L [IQR]	302 [231-383] n=34	491 [412-698] n=59	435 [294-591] n=93

COPD: Chronic Obstructive Pulmonary Disease, DLBCL: Diffuse Large B-Cell Lymphoma, HIV: Human Immunodeficiency Virus ILD: Interstitial Lung Disease, LDH: Lactate Dehydrogenase

Supplementary Table 3. Medication before admission (not related to COVID-19 treatment)

	**Ward (n=36)**	**ICU (n=61)**	**Total (n=97)**
Anti-hypertensive	20 (56%)	33 (54%)	53 (55%)
Statin	9 (25%)	22 (36%)	31 (32%)
NSAID use within 7 days of admission	3/9 (33%)	3/27 (11%)	6/36 (17%)
Anti-platelet	9 (25%)	12 (20%)	21 (22%)
Anti-coagulation	5 (14%)	1 (2%)	6 (6%)
Anti-diabetics	7 (19%)	9/60 (15%)	16/96 (17%)
Chronic systemic corticosteroid	0	4 (7%)	4 (4%)
Immunosuppressive medication (non-corticoid)	1 (3%)	5 (8%)	6 (6%)

NSAID: Non-steroidal anti-inflammatory drug

Supplementary Table 4. Ethnicity of COVID-19 patients

	**Ward (n=36)**	**ICU (n=61)**	**Total (n=97)**
Caucasian	30 (83%)	55 (90%)	85 (88%)
Black or sub-Sahara	2 (6%)	0	2 (2%)
Middle East	2 (6%)	0	2 (2%)
North African	2 (6%)	1 (2%)	3 (3%)
Latino or Hispanic	0	1 (2%)	1 (1%)
Not reported	0	4 (7%)	4 (4%)

Supplementary Table 5. Overview of used antibodies for flow cytometry

Antigen	Clone	Fluorochrome	Purchased from	Intracellular	Extracellular
anti-CD107a	eBioH4A3	AF488	Invitrogen	X	
anti-CD14	61D3	PerCP Cy5.5	eBiosciences	X	
anti-CD16	3G8	BUV395	BD biosciences/Horizon	X	
anti-CD3	SK7	APC-eF780	Invitrogen	X	
anti-CD42a	LMA.16	PE	BD biosciences/Horizon	X	
anti-CD49a	TS2/7	PE-Cy7	Biolengd	X	
anti-CD56	NCAM16.2	BV421	BD biosciences/Horizon	X	
anti-CD57	TB01	PE-Cy7	eBiosciences	X	
anti-CD62P	AK-4	APC	BD biosciences/Horizon	X	
anti-CD69	FN50	APC-R700	BD biosciences/Horizon	X	
anti-granzyme A	CB9	PE	Invitrogen		X
anti-granzyme B	GB11	BV510	BD biosciences/Horizon		X
anti-granzyme K	G3H69	eFF660	Invitrogen		X
anti-HLA-DR	G46-6	BV510	BD biosciences/Horizon	X	
anti-HLA-E	3D12	Pe-Cy7	Biolgend	X	
anti-IFN-γ	4S.B3	APC	Invitrogen		X
anti-KIR a/b/e1e2 (inhibitory)	CD158a - EB6B: CD158a/KIR2DL1 CD158b - GL183: CD158b1/KIR2DL2 CD158b2/KIR2DL3 CD158e1/e2 - Z27,3,7: CD158e1/KIR3DL1	PE	Beckman Coulter	X	
anti-KIR g/h (activating)	#1165A CD158g/KIR2DS5 #1127B CD158h/KIR2DS1	AF647	R&D	X	
anti-KIR-i (activating)	179315 CD158i/KIR2DS4	AF647	BD biosciences/Horizon	X	
anti-NKG2A	Z199	Pe-Cy7	Beckman Coulter	X	
anti-NKG2D	1D11	BV510	BD biosciences/Horizon	X	
anti-NKp30	p30-15	BV711	BD biosciences/Horizon	X	
anti-NKp46	9E2/NKp46	BV510	BD biosciences/Horizon	X	
anti-PD-1	EH12.1	BB515	BD biosciences/Horizon	X	
anti-PD-L1	MIH1	BUV395	BD biosciences/Horizon	X	
anti-perforin	deltaG9	AF488	Invitrogen		X
anti-TNF-α	Mab11	PE	Invitrogen		X

Supplementary Figures

Supplementary Figure 1

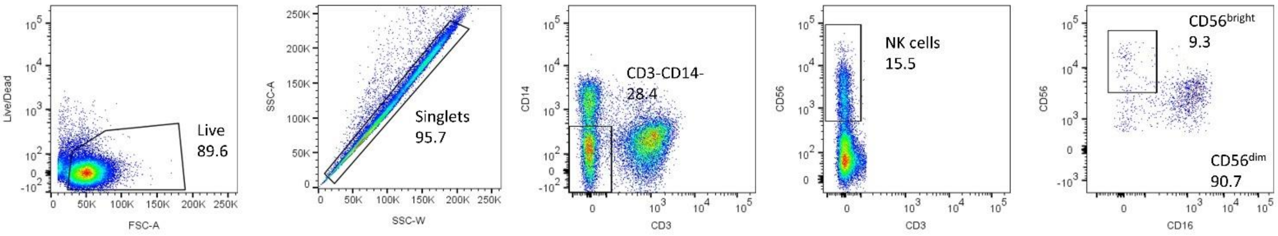

Supplementary Figure 1. Gating strategy for flow cytometry experiments.

Supplementary Figure 2

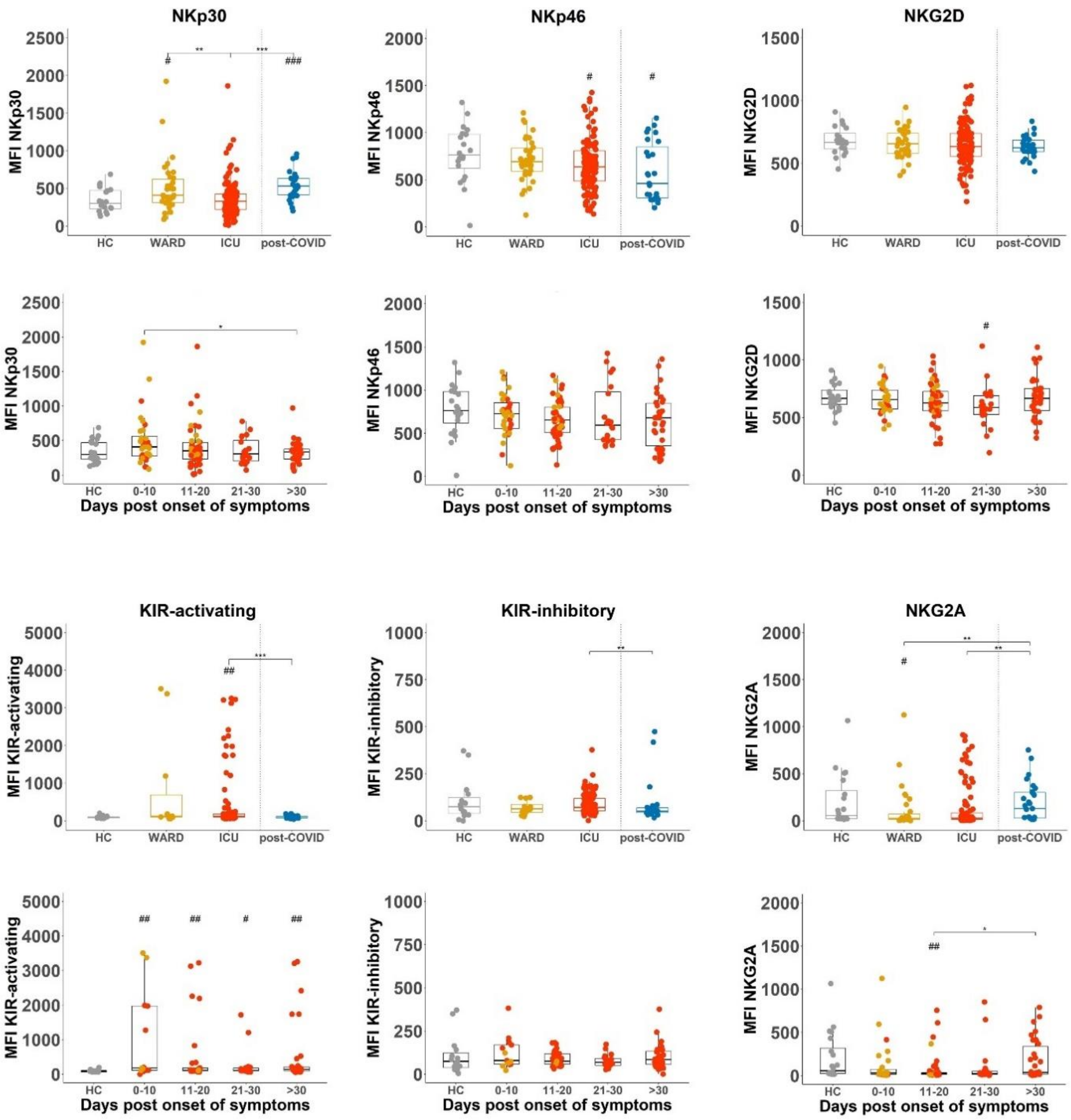

Supplementary Figure 2. Changes in the expression of activating and inhibiting NK cell receptors in COVID-19 WARD and ICU patients. Mean fluorescence intensity (MFI) of indicated activating and inhibitory receptors on natural killer (NK) cells of healthy controls (HCs) (n=18), WARD (n=34), ICU (n=61), and post-COVID-19 patients (n=28), upon return to the hospital six weeks after discharge. WARD (yellow) and ICU (red) samples were further subdivided based on the days post onset of COVID-19 symptoms. Each symbol represents a single patient. For the comparison of groups, p-values were obtained using Wilcoxon signed-rank test with Bonferroni correction. * Represent differences between indicated groups, # represent differences with HCs. */#p<0.05; **/##p<0.01; ***/###p<0.001

Supplementary Figure 3

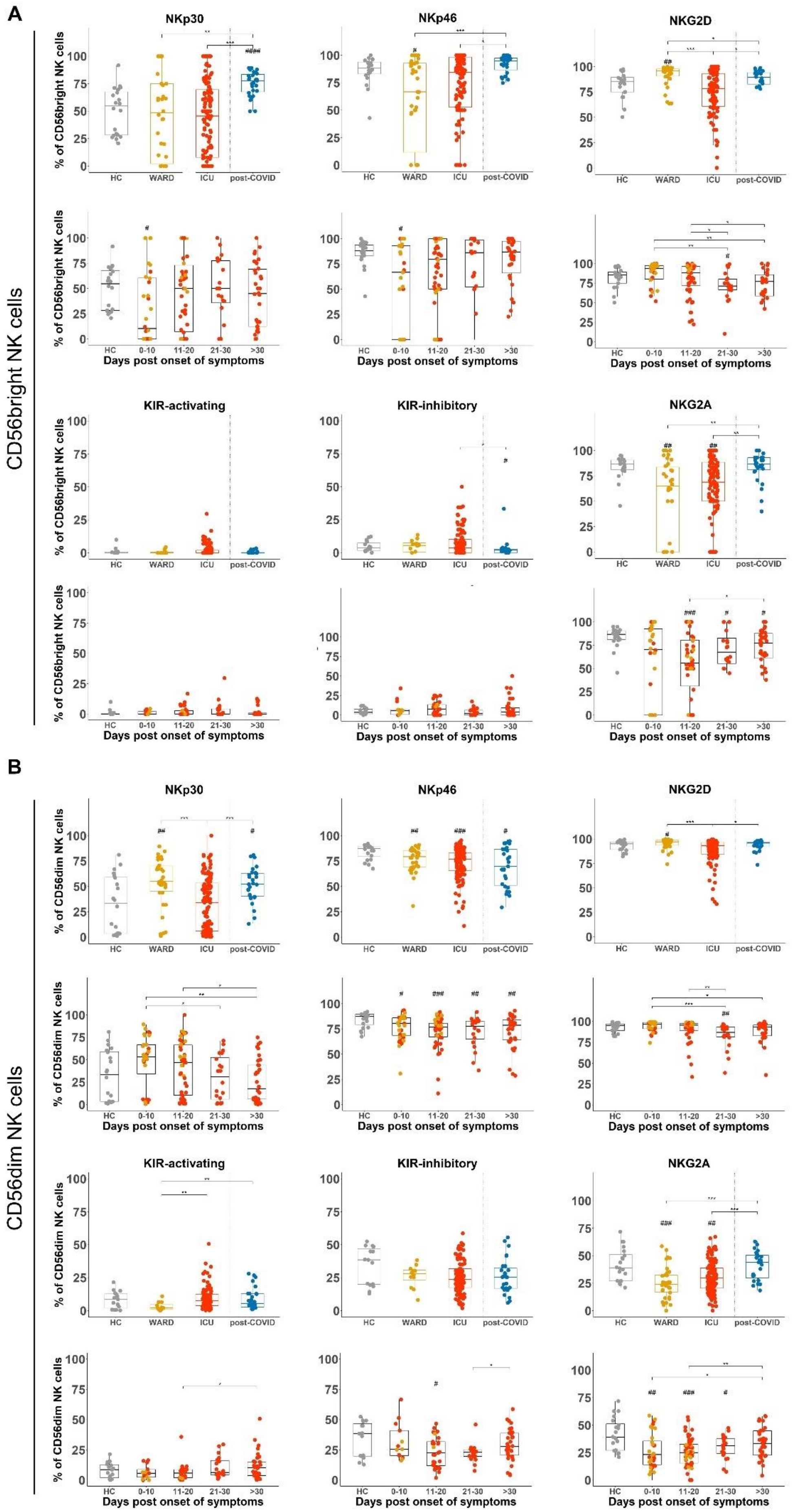

Supplementary Figure 3. COVID-19 patients present with a disturbed NK receptor profile. (A-B) Percentage expression of activating and inhibitory receptors on CD56^{bright} **(A)** and CD56^{dim} **(B)** natural killer (NK) cells of healthy controls (HCs) (n=18), WARD (n=34), ICU (n=61), and post-COVID-19 patients (n=28), upon return to the hospital six weeks after discharge. WARD (yellow) and ICU (red) samples were further subdivided based on the days post onset of COVID-19 symptoms. Each symbol represents a single patient. For the comparison of groups, p-values were obtained using Wilcoxon signed-rank test with Bonferroni correction. * Represent differences between indicated groups, # represent differences with HCs. */#p<0.05; **/##p<0.01; ***/###p<0.001; ****/####p<0.0001.

Supplementary Figure 4

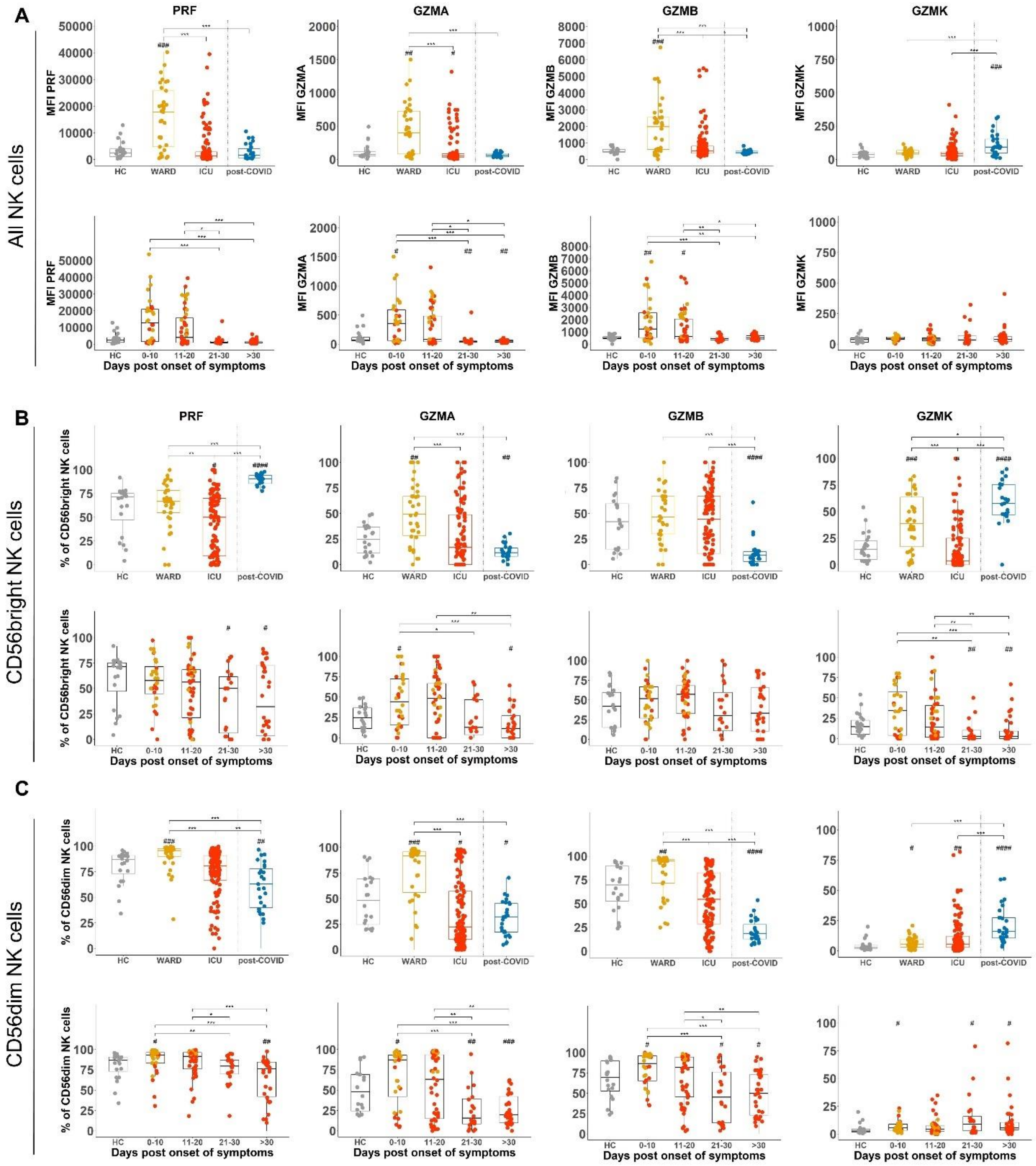

Supplementary Figure 4. Highly increased expression of cytotoxic molecules in WARD but not in ICU patients. (A) Mean fluorescence intensity (MFI) of indicated cytotoxic molecules on natural killer (NK) cells of healthy controls (HCs) (n=18), WARD (n=34), ICU (n=61), and post-COVID-19 patients (n=28), upon return to the hospital six weeks after discharge. WARD (yellow) and ICU (red) samples were further subdivided based on the days post onset of COVID-19 symptoms. **(B-C)** Percentage expression of cytotoxic molecules on $CD56^{bright}$ **(B)** and $CD56^{dim}$ **(C)** NK cells of indicated groups. Each symbol represents a single patient. For the comparison of groups, p-values were obtained using Wilcoxon signed-rank test with Bonferroni correction. * Represent differences between indicated groups, # represent differences with HCs. */#p<0.05; **/##p<0.01; ***/###p<0.001; ****/####p<0.0001.

Supplementary Figure 5

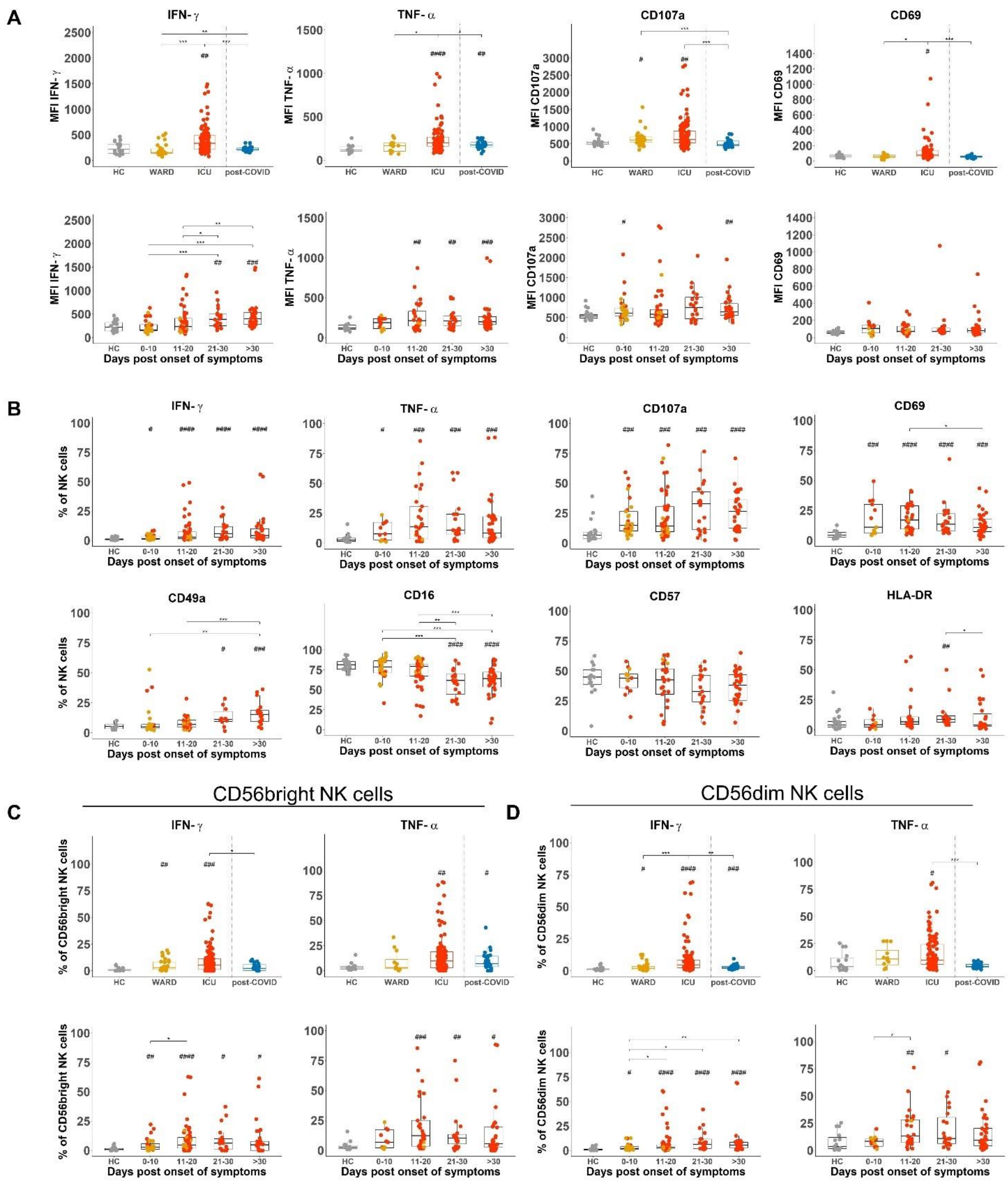

Supplementary Figure 5. Analysis of IFN-γ and TNF-α production by NK cells and association with the expression of CD107a, CD69, CD49a, CD16, CD57, and HLA-DR. (A) Mean fluorescence intensity (MFI) of indicated molecules on natural killer (NK) cells of healthy controls (HCs) (n=18), WARD (n=34), ICU (n=61), and post-COVID-19 patients (n=28), upon return to the hospital six weeks after discharge. WARD (yellow) and ICU (red) samples were further subdivided based on the days post onset of COVID-19 symptoms. **(B)** Percentage of indicated markers on natural killer (NK) cells of indicated patient groups. (**C-D**) Percentage of IFN-γ and TNF-α in CD56^{bright} **(C)** and CD56^{dim} **(D)** natural killer (NK) cells of indicated patient groups. Each symbol represents a single patient. For the comparison of groups, p-values were obtained using Wilcoxon signed-rank test with Bonferroni correction. * Represent differences between indicated groups, # represent differences with HCs. */#p<0.05; **/##p<0.01; ***/###p<0.001; ****/####p<0.0001.

Supplementary Figure 6

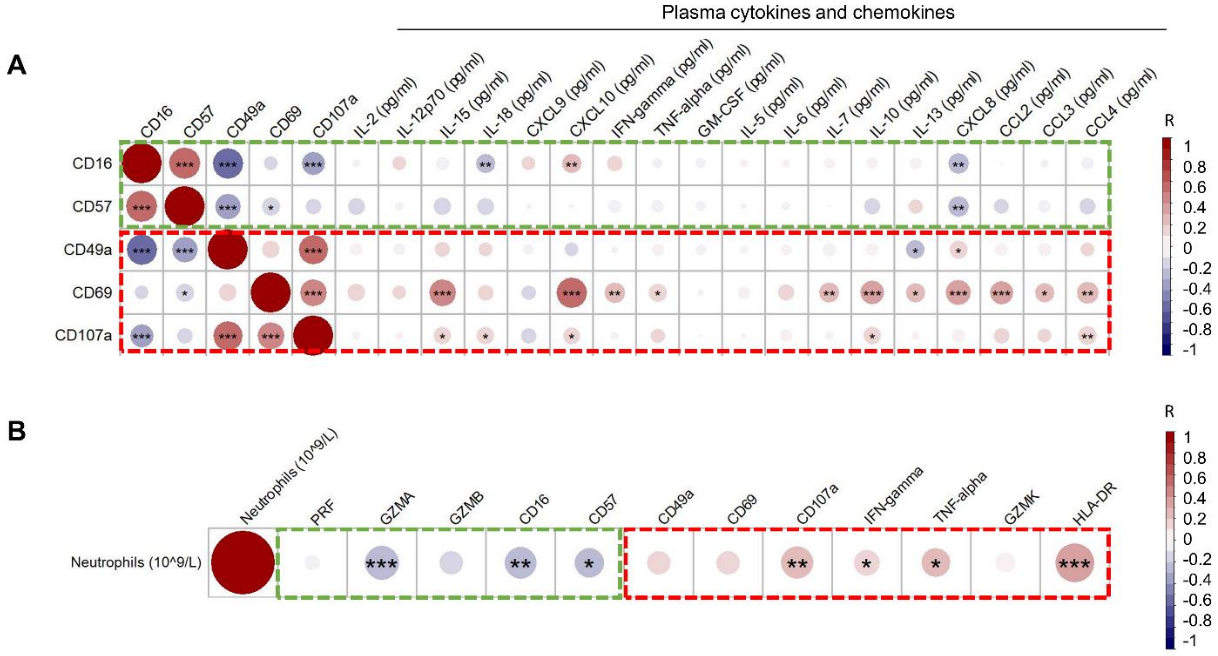

Supplementary Figure 6. Correlation of the activated NK cell subset with plasma cytokines and chemokines, and with the number of circulating neutrophils. (A) Correlation matrix for CD16, CD57, CD49a, CD69, and CD107a on natural killer (NK) cells and plasma levels of cytokines and chemokines. **(B)** Correlation matrix for perforin (PRF), granzyme A (GZMA), GZMB, CD16, CD57, CD49a, CD69, CD107a, IFN-γ, TNF-α, GZMK, and HLA-DR on NK cells, and the number of neutrophils. Pearson correlation was applied. * $p<0.05$; ** $p<0.01$; *** $p<0.001$.

Supplementary Figure 7

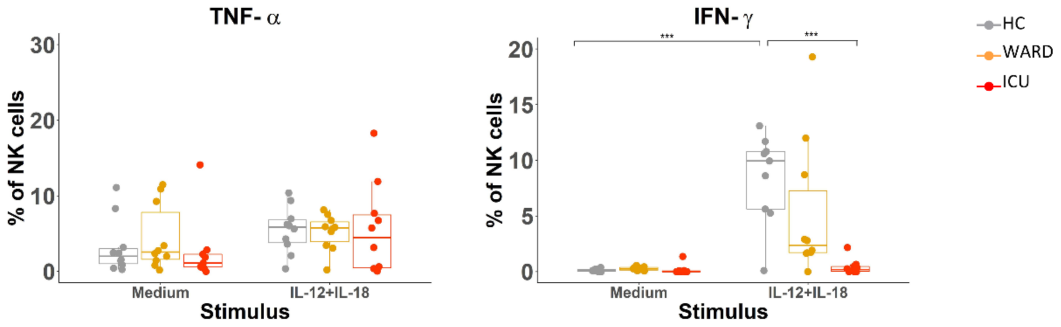

Supplementary Figure 7: Defective production of interferon-γ (IFN-γ) in natural killer (NK) cells from ICU COVID patients following stimulation with interleukin-12 (IL-12) and IL-18. Peripheral blood mononuclear cells from healthy controls (HCs) (n=10), WARD (n=10), and ICU (n=10) were cultured in medium alone or in the presence of IL-12 plus IL-18 for 18 hours. IFN-γ and tumor necrosis factor-α (TNF-α) was measured by intracellular flow cytometry. Each symbol represents a single patient. For the comparison of groups, p-values were obtained using Wilcoxon signed-rank test with Bonferroni correction. ***p<0.001 represent differences between indicated groups.

Supplementary Figure 8

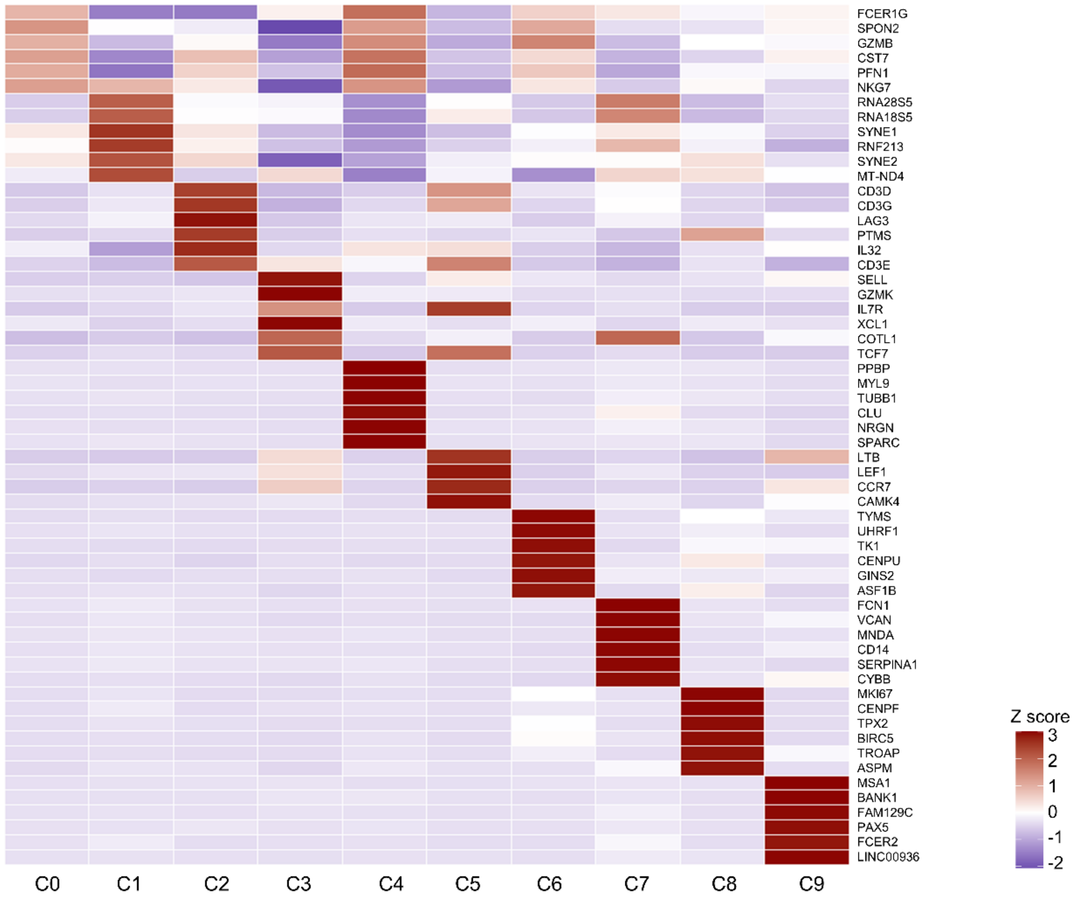

Supplementary Figure 8. Heatmap showing key marker genes of the distinct natural killer (NK) cell subsets.

Supplementary Figure 9

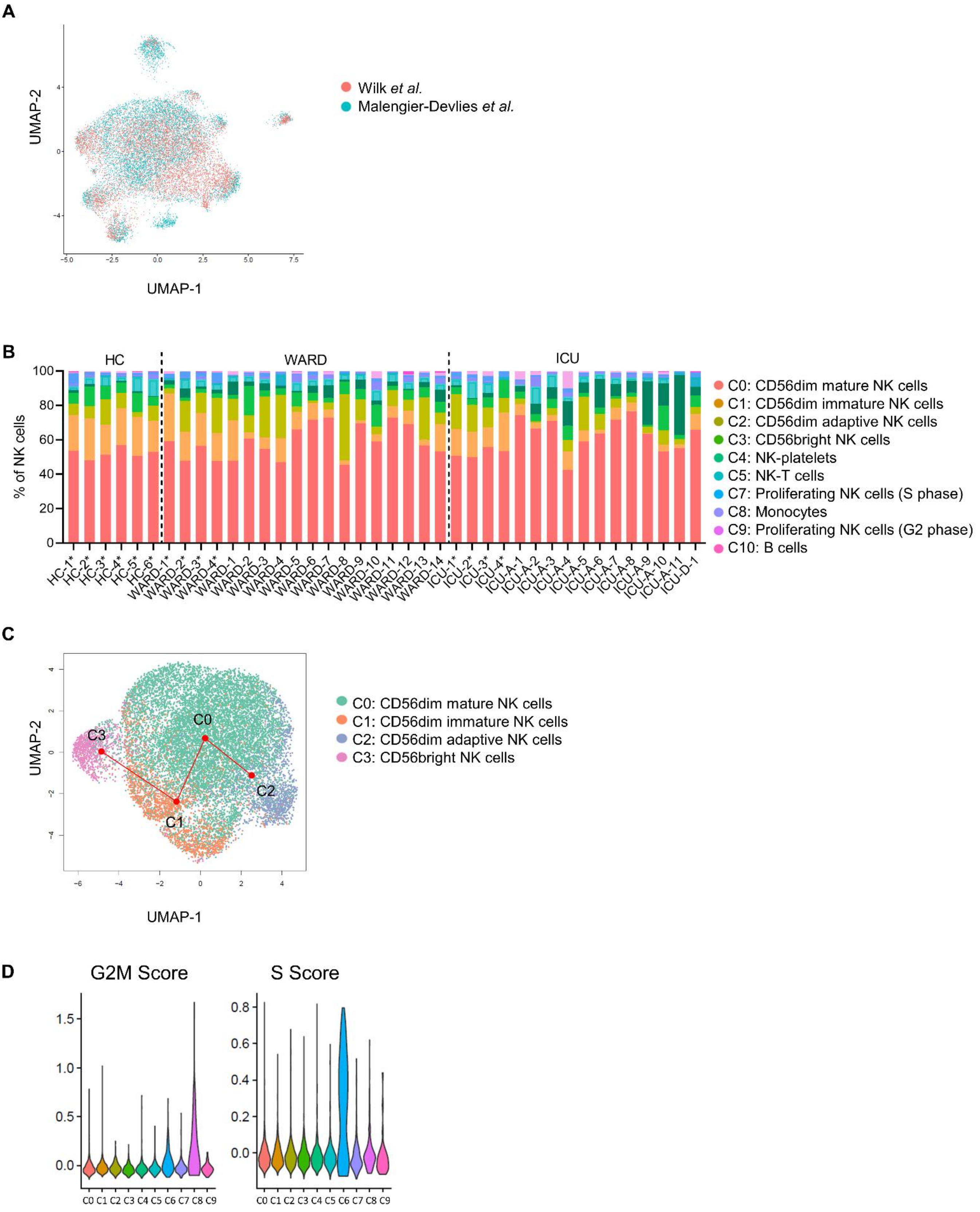

Supplementary Figure 9. Single-cell RNA-sequencing reveals 10 distinct NK cell-related subsets.

(A) UMAP presentation showing sub-clustered natural killer cells (NK) cells, stratified per dataset **(B)** The relative contribution of each NK cell type (in %) in each individual patient. **(C)** Pseudotime trajectory for NK cells (C0-C3) based on Slingshot. **(D)** Proliferation (G2M and S phase) was scored in the different NK cell subsets.

Supplementary Figure 10

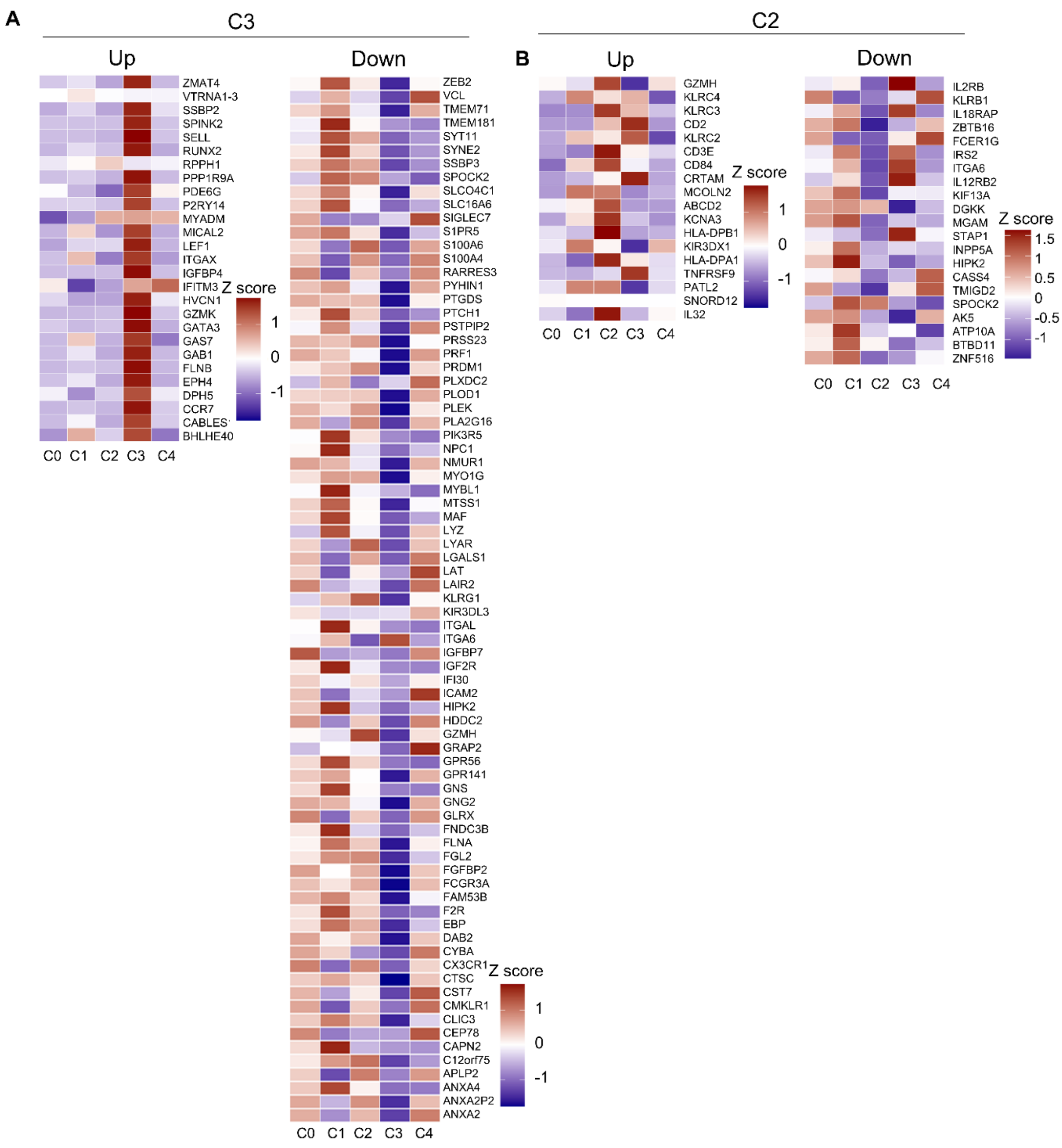

Supplementary Figure 10. Comparison of genes of the 5 NK cell clusters with published data.

(A) Heatmap of key up-and down-regulated CD56^{bright} natural killer (NK) cell markers according to Collins *et al.*. **(B)** Heatmap of key up-and down-regulated adaptive NK cell markers according to Schlums *et al.*.

Supplementary Figure 11

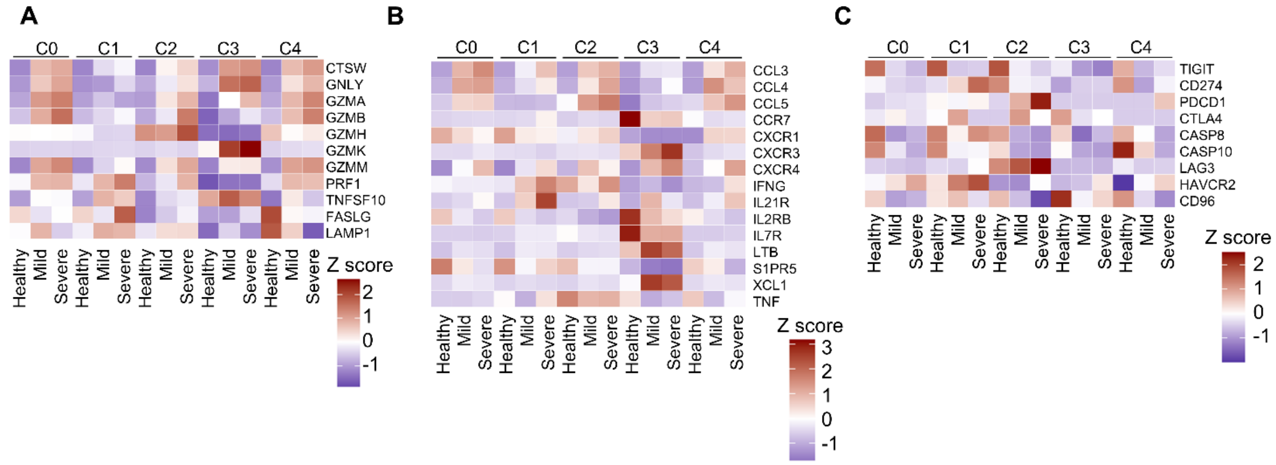

Supplementary Figure 11. Single-cell RNA-sequencing demonstrates an activated, non-exhausted NK cell phenotype in COVID-19 patients.

(A-C) Heatmap showing the expression of cytotoxic molecules **(A)**, cytokines and chemokines **(B)**, and exhaustion markers **(C)** in the different NK cell subsets of healthy controls (HCs), mild, and severe COVID-19 patients respectively.

Supplementary Methods

Subdivision of NK cell subsets by scRNA-seq in COVID-19 WARD and ICU patients, description of clusters C5-C9

C5 represents the NKT cells defined by the expression of *CD3*, *IL-7R*, *LEF1*, and *CCR7* (Supplementary Figure 9). Both C6 and C8 (1.5% and 1.2% of the total NK cells respectively) were annotated as proliferating cells. Gene signature analysis confirmed C6 to be the S phase NK cells, and C8 to be the G2 phase NK cells (Supplementary Figure 10D). Another small cluster, representing 1.5% of the total NK cells (C7), expressed i.e. *S100A12*, *CD14*, *MNDA*, *SERPINA1*, and *SPI1* and were annotated as monocytes (Supplementary Figure 9). Finally, a neglectable C9 (0.2% of the total NK cells) represented the B cells and expressed i.e. *MS4A1* (CD20), *BANK1*, and *PAX5* (Supplementary Figure 9). Both in C5, C6, C7, C8, and C9, no differences were found between HCs or COVID patients or between WARD and ICU patients (Figure 5B).
